# LOV Histidine Kinase Modulates the General Stress Response System and Affects the *virB* Operon Expression in *Brucella abortus*

**DOI:** 10.1371/journal.pone.0124058

**Published:** 2015-05-19

**Authors:** Gabriela Sycz, Mariela Carmen Carrica, Tong-Seung Tseng, Roberto A. Bogomolni, Winslow R. Briggs, Fernando A. Goldbaum, Gastón Paris

**Affiliations:** 1 Laboratorio de Inmunología y Microbiología Molecular, Fundación Instituto Leloir (IIBBA-CONICET), Buenos Aires, Argentina; 2 Department of Plant Biology, Carnegie Institution for Science, Stanford, California, United States of America; 3 Department of Chemistry and Biochemistry, University of California Santa Cruz, Santa Cruz, California, United States of America; East Carolina University School of Medicine, UNITED STATES

## Abstract

*Brucella* is the causative agent of the zoonotic disease brucellosis, and its success as an intracellular pathogen relies on its ability to adapt to the harsh environmental conditions that it encounters inside the host. The *Brucella* genome encodes a sensor histidine kinase containing a LOV domain upstream from the kinase, LOVHK, which plays an important role in light-regulated *Brucella* virulence. In this report we study the intracellular signaling pathway initiated by the light sensor LOVHK using an integrated biochemical and genetic approach. From results of bacterial two-hybrid assays and phosphotransfer experiments we demonstrate that LOVHK functionally interacts with two response regulators: PhyR and LovR, constituting a functional two-component signal-transduction system. LOVHK contributes to the activation of the General Stress Response (GSR) system in *Brucella* via PhyR, while LovR is proposed to be a phosphate-sink for LOVHK, decreasing its phosphorylation state. We also show that in the absence of LOVHK the expression of the *virB* operon is down-regulated. In conclusion, our results suggest that LOVHK positively regulates the GSR system *in vivo*, and has an effect on the expression of the *virB* operon. The proposed regulatory network suggests a similar role for LOVHK in other microorganisms.

## Introduction

*Brucella* spp. are Gram-negative bacteria that belong to the α-2-proteobacteria group. They are responsible for one of the world’s most widespread zoonotic infections, brucellosis, causing miscarriage and infertility in animals and a febrile disease in humans known as Malta or undulant fever [1, 2]. *Brucella* is a facultative intracellular pathogen that can infect professional and non-professional phagocytes. After internalization, bacteria persist and replicate inside a modified vacuole called the *Brucella*-containing vacuole (BCV), a compartment with membranes derived from the endoplasmic reticulum [3]. During intracellular trafficking and active replication inside the BCV, *Brucella* cells encounter low oxygen tension, low pH, reactive oxygen and nitrogen species, and nutrient deprivation. The primary basis for the tenacity of *Brucella* infections lies in their capacity to persist for prolonged periods in the phagosomal compartment of these phagocytes. In order to survive under these extreme conditions, *Brucella* is able to sense and respond to environmental factors by appropriately modifying RNA and protein expression [4, 5].

*Brucella*, like many other bacteria, can sense and respond to environmental changes through two-component systems (TCS), which typically consist of a sensor histidine kinase (HK) and its cognate response regulator (RR). Usually, sensor HKs are proteins consisting of a sensor domain at the N-terminal end, linked to a C-terminal HK domain. Activation of the sensor domain by a chemical or physical signal leads to an increase in autophosphorylation of a His residue in the HK domain. Subsequently, the phosphate is transferred to a conserved Asp residue in the receiver (REC) domain of the RR protein [6]. Some RRs only have a REC domain, and others have a C-terminal effector domain which could be a DNA or protein-binding domain, or could have enzymatic activity. This effector domain elicits an output response, which finally modulates gene expression and cellular physiology [7].

*Brucella* spp. genomes encode a LOV-domain-containing protein that is associated to a PAS (Per, Arnt and Sim) domain plus a HK domain (LOVHK), which belongs to the HWE family [8]. LOV domains are blue-light sensory modules which bind a flavin molecule as cofactor [9]. Activation of LOVHK by blue-light increases the autophosphorylation activity of the HK domain. LOVHK plays a role in the intracellular survival of *Brucella*, since deletion of this gene in *B*. *abortus* shows an attenuated phenotype in cell-culture infection assays [10, 11].

Light is the main source of energy of the biosphere but it also has damaging effects on many biological molecules. Many bacteria have evolved photoreceptor proteins that sense light and in particular blue-light, to elicit adaptive responses to this stress factor. LOV-domain-containing proteins from other bacteria have been associated with stress responses. For example, light perception through LOV proteins has been associated with activation of stress responses in the Gram-positive bacteria *Bacillus subtilis* [12] and *Listeria monocytogenes* [13]. Recently, a relationship between LOV proteins and the RR, PhyR, has been shown in two members of the α-proteobacteria group: *Caulobacter crescentus* CB15 and *Erythrobacter litoralis* HTCC2594. While in *C*. *crescentus* the interaction between a LOV protein and PhyR was demonstrated using a genetic approach [14], in *E*. *litoralis* this relationship was shown by phosphotransfer assays *in vitro* [15]. LOV proteins of both species not only interact with PhyR but they are also able to interact with single-domain response regulators [15, 16].

PhyR is the main regulator of the General Stress Response (GSR) system that protects bacteria against a wide variety of stress conditions. This system is conserved within and restricted to all α-proteobacteria [17], and has been described in *Methylobacterium extorquens* AM1 [18, 19], *Bradyrhizobium japonicum* USDA 110 [20], *C*. *crescentus* CB15 [21–23], *Sphingomonas* sp. Fr1 [24], *Sinorhizobium meliloti* [25, 26], *Bartonella quintana* [27], *Methylosinus* sp. B4S [28], *Rhizobium etli* [29], *Rhodopseudomonas palustris* TIE-1 [30], and *Brucella abortus* 2308 [31]. The GSR system consists of an alternative extracytoplasmic function (ECF) sigma factor known as RpoE that, in the absence of stress or a particular stimulus, binds the anti-sigma factor NepR and maintains the system in an inactive state. In the presence of a stimulus such as peroxides, increase of osmotic pressure, UV light, or desiccation, the anti-sigma factor is sequestered by PhyR allowing the ECF sigma factor to associate with the core RNA polymerase, and direct the expression of specific genes in response to stress. PhyR is a RR that has an N-terminal sigma factor-like domain which binds NepR, and a C-terminal REC domain with a conserved Asp residue [17]. *In vitro*, phosphorylation of this residue using acetyl-phosphate or beryllium trifluoride is required for PhyR/NepR interaction in many α-proteobacteria [19, 32, 33]. In *B*. *abortus*, unphosphorylated PhyR and RpoE1 have similar interaction affinities with NepR. However, phosphorylation of PhyR increases its affinity for NepR, thus stabilizing PhyR-NepR complexes. Interestingly, *in vivo*, in the absence of stress PhyR protein is degraded by the ClpXP protease. In the presence of stress, PhyR protein level is stabilized by inactivation of ClpXP protease and/or PhyR phosphorylation [31]. Although this system responds to different stress factors, up to now a HK able to phosphorylate PhyR has not been described in *Brucella* spp.

In the present work, using a combination of two-hybrid assays and phosphotransfer experiments we identify two response regulators as partners of LOVHK and provide further information on the LOVHK-associated regulatory network. The results presented here increase the understanding of the role of this signaling pathway in *Brucella* virulence.

## Materials and Methods

### Bacterial strains and culture conditions

Genetic manipulations were carried out in *Escherichia coli* DH5α in Luria-Bertani medium (LB, Difco) at 37°C on a shaker at 250 rpm with the appropriate antibiotics added. *E*. *coli* BL21(DE3)pLysS was used for protein expression and was grown in autoinducer medium ZYM-5052, MD-5052 medium or in LB [34]. *E*. *coli* S17-1 was used for conjugation with *B*. *abortus*. When used, antibiotics were added to the following final concentrations: 100 μg ml^-1^ ampicillin, 25 μg ml^-1^ kanamycin, 12.5 μg ml^-1^ tetracycline, 25 μg ml^-1^ chloramphenicol.

*Brucella abortus* 2308 was grown at 37°C on a shaker set at 250 r.p.m. in rich medium Tryptic Soy Broth (TSB, Difco) from an initial OD_600nm_ of 0.05, or in Tryptic Soy Agar (TSA, Difco). When used, antibiotics were added to the following final concentrations: 50 μg ml^-1^ ampicillin, 25 μg ml^-1^ kanamycin, 10 μg ml^-1^ nalidixic acid, and 20 μg ml^-1^ chloramphenicol for agar plates and 5 μg ml^-1^ chloramphenicol for liquid cultures. All *Brucella* strains used in this study were manipulated in a biosafety level 3 laboratory available at Leloir Institute following national regulations. All *E*. *coli* and *Brucella* strains used in this study are listed in S1 Table.

### DNA manipulations

DNA manipulations were performed according to standard techniques. PCR reactions were performed using *B*. *abortus* 2308 genomic DNA as template and Pfx polymerase (Invitrogen) according to the manufacturer’s instructions. The amplified products were digested with the indicated restriction enzymes and cloned into the same restriction sites of the corresponding plasmids. All plasmids and primers used are listed in S1 Table and S2 Table, respectively.

#### Cloning of the *lovhk* and *pdhS* genes for the two hybrid assays

The *lovhk* (BAB2_0652) gene was amplified using primers Full_LOV_forward and Full_LOV_reverse (S2 Table). The 1.4 Kb PCR product obtained was cloned into pBT plasmid (BacterioMatch II Two-Hybrid System Kit, Stratagene) using NotI and XhoI. The resulting pBT construct expresses the *lovhk* gene in frame with the λcl gene. The *pdhS* gene was also cloned into pBT plasmid to be used as a positive-interaction control using primers PdhS_c-ter_forward and PdhS_c-ter_reverse [35].

#### Cloning of RR genes for the two hybrid assays

The *B*. *abortus* 2308 genome has 24 genes encoding proteins containing REC domains (S3 Table). All REC domains were cloned except for the gene that corresponds to a hybrid HK (BAB1_0346) and a pseudogene (ΨBAB1_1059). Primers listed in S2 Table were used to amplify each gene. All forward oligonucleotides had the NotI restriction site, except for the BAB2_0630_forward that had an EcoRI restriction site. All reverse oligonucleotides had an XhoI restriction site. The PCR products digested with the corresponding restriction enzymes were cloned into the pTRG plasmid (BacterioMatch II Two-Hybrid System Kit, Stratagene) to obtain constructs in-frame with the RNAPα gene (pTRG). A minilibrary of REC-containing genes was created by mixing equal amounts of pTRG constructs.

#### Cloning of *Brucella* genes for recombinant protein expression

*lovhk* gene (BAB2_0652) was amplified with primers LOVHK_Full_NheI_Fw and LOVHK_Full_XhoI_Rev, containing the NheI and XhoI restriction sites respectively. The *hk* domain of *lovhk* was amplified with primers BaLOV_5Dhpt_NheI and BaLOV_3CAL_SalI. The *lovR* gene (BAB1_0099) was amplified with primers LovR_FWD_NheI and LovR_REV_XhoI. The *phyR* gene (BAB1_1671) was amplified with primers 1671_NdeI_FWD and 1671_His_Stop_REV. The *lovhk* and *hk* domain were cloned into pET24a (Novagen) expression plasmid, using the corresponding restriction enzymes. *phyR* gene was first cloned into pGEM-T Easy (Promega) generating the plasmid pGEM-PhyR, and subsequently subcloned into pET24a (Novagen) expression plasmid, using NdeI and NotI restriction enzymes. *lovR* was cloned into the pTrcHisB (Invitrogen) expression plasmid using the corresponding restriction enzymes. All primers are listed in S2 Table. All the constructions were sequenced to confirm the absence of mutations. Plasmids expressing the different recombinant proteins were transformed into BL21 (DE3) pLysS.

### Bacterial two-hybrid assays

Two-hybrid assays were carried out using the BacterioMatchII two-hybrid system (Stratagene) following the manufacturers’ instructions. This system uses an *E*. *coli* reporter strain (BacterioMatch II Two-Hybrid System Reporter Cells), and detects protein-protein interactions based on transcriptional activation of the His3 gene. The reporter cells were co-transformed with the corresponding plasmids and incubated at 28°C from 24 to 48 h until colonies were visible. Interactions were determined to be positive by growth on selective screening medium containing minimal M9 medium plus 5 mM 3-amino-1,2,4-triazole (3-AT), 25 μg ml^-1^ chloramphenicol and 12.5 μg ml^-1^ tetracycline, and validated by growth in dual selective screening minimal M9 medium plus 5 mM 3-AT and 12.5 μg ml^-1^ streptomycin, 25 μg ml^-1^ chloramphenicol, and 12.5 μg ml^-1^ tetracycline (double positive clones). LOVHK was used as bait and a library with the 23 RR as prey. For the second two-hybrid assay, LOVHK was used as bait and a library containing 22 RRs (LovR was not included) as prey. In order to identify to which RR the double positive colonies corresponded to, the inserts were amplified by PCR and sequenced. Protein interactions in the two-hybrid assays were quantified using the FW102 O_L_2-62 *E*. *coli* reporter strain [36] as indicated in Carrica *et al*., 2012 [38], except that protein expression was induced for 3 h. Interactions with the corresponding empty vectors were used as negative controls, and the interaction between strains co-transformed with pBT-GF2 and pTRG-Gal11 plasmids was used as a positive control. Both experiments were conducted under normal light conditions, and β-galactosidase activity was assayed using a standard Miller protocol.

### Expression and purification of recombinant proteins

For LOVHK protein purification, culture was grown in minimal autoinducer medium (MD-50529) and only in this case all culture manipulations, protein purification and incubations were carried out under dark or dim red light conditions. *E*. *coli* cells expressing HK or PhyR recombinant proteins were grown in rich autoinducer medium (ZYM-5052) at 37°C for 3 h, and then incubation was continued at 18°C for 16 h. Cells were harvested by centrifugation at 8,000 x g for 20 min and resuspended in lysis buffer (20 mM Tris-HCl pH 8.0, 500 mM NaCl, 20 mM imidazole, 0.05% Triton X-100 and 1 mM phenylmethylsulphonylfluoride—PMSF), and disrupted by sonication with a probe tip sonicator (QSonica, LLC, Misonix XL-2000 series). The total cell lysates were centrifuged at 35,000 x g for 15 min and supernatants were incubated with nickel nitrilotriacetic acid-agarose resin (Ni-NTA agarose, Quiagen) with gently agitation at 4°C for 1 h. The resin was packed in a column, washed twice with washing buffer (20 mM Tris-HCl pH 8.0, 500 mM NaCl, 20 mM imidazole), and proteins were eluted with 5 ml of elution buffer (20 mM Tris-HCl pH 8.0, 500 mM NaCl, 200 mM imidazole). The fractions containing the corresponding proteins were dialyzed against dialysis buffer (20 mM Tris-HCl pH 8.0, 100 mM NaCl, 1 mM EDTA, 1 mM PMSF) and stored at 4°C until use. LovR was purified under denaturing conditions. Bacteria were grown in LB medium at 37°C for 3 h. Protein expression was induced with 1 mM isopropyl-thio-β-D-galactopyranoside (IPTG) and incubation was continued for 16 h at 18°C. Cells were harvested by centrifugation at 8,000 x g for 20 min, resuspended in lysis buffer with 8 M urea added, and disrupted by sonication. The purification protocol was continued as above with 8 M urea added to all buffers. Purified LovR was refolded by dialysis against dialysis buffer at 4°C, and then stored at 4°C until use. The purification procedures of all proteins were evaluated in 12–15% SDS-PAGE and stained with Coomassie Brilliant Blue. The PAS-HK-NtrY recombinant protein was expressed and purified as previously described [38] and the recombinant REC domain of NtrX was a kind gift of Ignacio Fernández. For anti-PhyR antibody production, PhyR recombinant protein was further purified in a Superdex-75 gel filtration column (GE healthcare) equilibrated with buffer 20 mM Tris-HCl pH 8.0, 300 mM NaCl. The samples containing PhyR were pooled and concentrated by centrifugation using a centrifuge filter (Millipore).

### Phosphotransfer assays

Purified LOVHK protein at a concentration of 2.5 μM was irradiated with 10 flashes of white light (a fluence of 4000 umol m-2 sec-1 of a xenon flashlamp) in phosphorylation buffer (20 mM Tris-HCl pH 8, 50 mM NaCl, 5 mM MgCl_2_, 100 μM ATP) containing 10 μCi of [γ-^32^P] ATP (111TBq/mmol, PerkinElmer Life Sciences). After 15 min at 37°C, purified RRs were added to the mixture to a final concentration of 2.5 μM each (final volume: 100ul). At the indicated times, aliquots of 10 μl were drawn and the reaction was stopped with an equal volume of 2X Laemmli sample buffer. Incubations were carried out under regular white light room illumination. Samples were separated by 15% SDS-PAGE, dried, exposed to a Storage Phosphor Screen (GE Healthcare), and scanned by a Storm 840 Molecular Imager (GE Healthcare) or exposed to a radiographic film. The intensity of each band was estimated using the ImageQuant 5.2 program (Molecular Dynamics) or Quantity One 4.6.3 Bio-Rad program. The relative intensity of each band to total intensity at time 20 seconds is reported in figures.

### Mutant-strain construction and complementation

The *B*. *abortus* 2308 *lovhk*::*km* mutant strain was previously obtained by a kanamycin 00CAssette insertion [10]. *∆lovR* and *∆phyR* mutant strains were obtained by clean deletion of those genes in *B*. *abortus* 2308 wt. Briefly, the 5´ and 3´ flanking regions of each gene were amplified with primers indicated in S2 Table, and both fragments (containing complementary regions) were ligated by overlapping PCR using the flanking oligonucleotides. The resulting fragment was cloned into plasmid pk18mobsacB [39] obtaining plasmids pk18mobsacB_*∆lovR* and pk18mobsacB_*∆phyR* respectively, which do not replicate in *Brucella*. Plasmids were transformed into *E*. *coli* S17-1 and transferred to *B*. *abortus* 2308 by conjugation. Single recombinants were selected by resistance to kanamycin and sensitivity to 10% sucrose in TSA plates. Double recombination events were selected by sensitivity to kanamycin and resistance to 10% sucrose in TSA plates. The excision of the plasmid and the generation of the mutant strains (named *∆lovR* and *∆phyR*, respectively) by allelic exchange were confirmed by colony PCR and sequencing of PCR products.

For complementation assays, the *lovhk*::*km* mutant strain was conjugated with pMR10*cat*_*lovhk* vector, which expresses *lovhk* under the control of its own promoter, obtaining the complemented strain *lovhk*::*km*/pMR_*lovhk*. The pMR10*cat*_*lovhk* vector was constructed by restriction free cloning method [40]. Briefly, the *lovhk* gene with a fragment of 581 bp upstream of the start codon was amplified with primers pMR10_pLOVHK_F and pMR10_pLOVHK_R (S2 Table), and the resulting fragment was used as a mega-primer for a PCR using pMR10*cat* vector as template, obtaining the pMR10*cat*_*lovhk* vector. This plasmid was transformed into *E*. *coli* S17-1 and transferred to *lovhk*::*km* mutant strain by conjugation. Bacteria bearing the pMR10*cat*_*lovhk* plasmid were selected by resistance to nalidixic acid, kanamycin and chloramphenicol in TSA plates. The presence of the plasmid was confirmed by PCR. The complemented strain showed a slower growth in TSB compared to the wt and *lovhk*::*km* mutant strains (S7 Fig). This effect is probably due to the expression of *lovhk* gene from a multicopy plasmid, which could be toxic as it has been previously reported for other HKs [41].

### Total RNA isolation from *Brucella*

*Brucella abortus* 2308 wt and the mutant and complemented strains were grown in TSB medium at 37°C. An aliquot of bacterial culture in logarithmic phase was mixed with 1/10 volumes of stop solution (ethanol:phenol, 19:1) and left at room temperature for 2 minutes. Cells were harvested by centrifugation at 10,000 x g for 2 min, the supernatant was removed, and total RNA isolation was carried out with MasterPure RNA Purification Kit (Epicentre, Illumina) following the manufacturer’s instructions, with some modifications. The pellets were resuspended in 500 μl of 1X T&C lysis solution (containing 1 μl Proteinase K at 50 μg μl^-1^ per 300 μl of 1X T&C lysis buffer), incubated at 65°C for 30 min with vortexing every 5 min, and then placed on ice for 5 min. Then, 0.25 ml of MPC solution was added to each tube and centrifuged at 14,000 rpm at 4°C for 10 min. The supernatants were mixed with 0.75 ml of isopropanol, and stored at -20°C for 16 h to 3 days. The samples were then centrifuged at 14,000 rpm at 4°C for 10 min. Pellets were air-dried and resuspended in 195 μl of DNAse buffer with 5 μl of DNaseI RNAse-Free, and incubated at 37°C for 40 min. Then, 0.2 ml of 2X T&C lysis solution was added to each tube and vortexed. 0.2 ml of MPC solution was added, vortexed again and placed on ice for 5 min. Samples were then centrifuged at 14,000 rpm at 4°C for 10 min. Then 0.5 ml of isopropanol was added to the supernatant and centrifuged at 14,000 rpm at 4°C for 10 min. The pellets were washed twice with 70% ethanol. Pellets were air-dried and then resuspended in 30 μl of RNase-free water. RNA was quantified using a Nano-Drop spectrophotometer (ND-1000, Thermo Fisher Scientific). DNA was subsequently removed by digestion with RQ1 RNase-free DNase (Promega) according to the manufacturer’s instructions.

### Real Time quantitative RT-PCR assays

Reverse transcription was performed with SuperScript III First Strand Synthesis System (Invitrogen) following the manufacturer’s instructions using random decamer primers (Invitrogen) and RNasin ribonuclease inhibitor (Promega). The cDNA samples obtained were used as templates in qRT-PCRs. Primers used in the reactions were designed with Primer Express v3.0.1 3 (Applied Technologies) and adjusted by visual inspection (S2 Table). qRT-PCR reactions were performed with SYBR Green in 96-well plates in an Mx3005P instrument (Stratagene) or in a StepOnePlus instrument (Applied Technologies), and analyzed with MxPro3005P or StepOne programs respectively. The results of each target were normalized to *B*. *abortus* Translation Initiation Factor-1 (*if-1*, BAB1_0282) mRNA [42] (S2 Table), and are presented as log_2_ of fold induction relative to wt.

### Antibody production

Specific antiserum against PhyR was raised in mice. Five mice were immunized on days 0, 15, 30 and 45 with 100 μg of PhyR recombinant protein mixed with Incomplete Freund’s Adjuvant (Sigma-Aldrich). After the fourth dose, mice were sacrificed and total blood was extracted. Anti-PhyR titer was determined by ELISA, and serum was stored at -20°C until use. All research involving animals has been conducted according to the National Institutes of Health Guide for the Care and Use of Laboratory Animals, and all experimental protocols have been approved by the Institutional Animal Care and Use Committee (IACUC) of Leloir Institute (Protocol number: 2009/08/41/FG).

### Western Blot analysis

*B*. *abortus* cells were grown in TSB up to logarithmic phase, and equal amounts of bacteria of each strain were harvested by centrifugation for 2 min at 10,000 x g, resuspended in Laemmli sample buffer and inactivated by heating at 100°C for 20 min. These samples were then separated on a 15% SDS-PAGE and transferred to a nitrocellulose filter (Millipore). RibH1, a lumazine synthase (LS) isoenzyme, was used as loading control as it exhibits constitutive expression in *B*. *abortus* [43, 44]. Membranes were probed with primary polyclonal mouse antiserum anti-PhyR (1:5,000), or polyclonal rabbit antiserum anti-RibH1 (1:2,000) in PBS-Tween 0.05% and 1% milk, with gently agitation at 4°C for 16 h. Membranes were then incubated with secondary antibody HRP-conjugated goat anti-mouse (1:2,000) (Sigma A4416) or HRP-conjugated goat anti-rabbit (1:5,000) (Sigma A6154), with gently agitation at room temperature for 1 h. Blots were developed using Pierce ECL Plus Western Blotting Substrate (Thermo Scientific), following the manufacturer’s instructions. Signal intensity was measured using a Storm 840 Molecular Imager (GE Healthcare), and then quantified using the ImageQuant 5.2 program.

### Stress induced by starvation

*Brucella abortus* 2308 wt and mutant strains were grown in TSB medium at 37°C, up to logarithmic phase (OD_600_ ≈ 1–1.5). An aliquot of each bacterial culture was taken (time 0 h), and the rest of the culture was washed once with a modified MM1 minimal medium [44] (57.3 mM K_2_HPO_4_, 35.9 mM KH_2_PO_4_, 0.1% w/v yeast extract, pH 7.0), and resuspended in the same medium. The cultures were incubated in a shaker at 37°C, and aliquots were drawn at 30 minutes, 1 h and 2 h. Total RNA was isolated from each sample and retro-transcribed as previously described. *phyR* mRNA expression was analyzed by qRT-PCR as previously described.

### PhyR stability

This experiment is based on an experiment conducted by Kim *et al*., 2013 [31], with some modifications as follows. *Brucella abortus* 2308 wt, *lovhk*::*km* and *∆lovR* mutant strains were grown in TSB medium at 37°C up to logarithmic phase (OD_600_ ≈ 1). An aliquot of each bacterial culture was taken (time 0 h) and then, in order to inhibit general protein synthesis, chloramphenicol was added to a final concentration of 500 μg ml^-1^. Cultures were incubated in a shaker at 37°C, and aliquots were drawn at 3 h and 5 h. PhyR protein level was analyzed by Western Blot. Relative protein level to time 0 h is shown for each strain.

### *VirB* promoter activity

The *virB* promoter was amplified using primers pvirbup and pvirbdown [45], and cloned into pBBR-*lacZ* vector (a pBBR1MCS-4_Amp^R^ vector containing the *lacZ* promoter-probe cassette) generating the plasmid pBBR-prom-*virB*-*lacZ*, where the *virB* promoter is fused to the *lacZ* reporter gene. This replicative plasmid was electroporated in *B*. *abortus* wt, *lovhk*::*km*, *ΔlovR* and *ΔphyR*. For complementation assays, the pBBR-prom-*virB*-*lacZ* plasmid was introduced by conjugation into the *lovhk*::*km/*pMR_*lovhk* complemented strain. The pMR10*cat* and pBBR1 based vectors are mobilized by RP4 and compatible with each other. Empty pBBR-*lacZ* was introduced in the wt 2308 strain and used as negative control. For promoter activity determination, two colonies from each strain were analyzed. Bacteria were grown in TSB from logarithmic to stationary phase, and two aliquots from each culture were drawn at each time (S6 Fig). β-galactosidase activity was determined in each sample as previously described [46], with the following modification: the reaction mixture was centrifuged before A_420_ determination. β-galactosidase activity is expressed in Miller Units [*A*_420_/(time x volume x OD_600_) x 1000]. This experiment was repeated three times.

### Statistical analysis

Data are presented as mean ± standard error or standard deviation of the mean. Significance is reported using one-way ANOVA followed by the appropriate post-hoc test as indicated in the legend of each figure. All analysis was performed using the R statistical package.

## Results

### Identification of two cognate RRs for LOVHK

Two-component systems usually consist of two proteins: a sensor domain linked to a HK domain and a RR containing the receiver and output domains [6]. Many HK and RR proteins are encoded in the same operon, evidence suggesting their functional interaction [47]. In other cases, the HK and the RR are actually encoded as a single protein and are named hybrid HKs [e.g. the LOV-HK-RR protein in *Pseudomonas syringae*, [48]].

In *Brucella*, LOVHK is encoded as an orphan histidine kinase with no obvious RR immediately downstream or upstream. In order to determine the identity of the RR functionally interacting with LOVHK, we carried out a bioinformatic analysis. Searches in *Brucella* genomic databases allowed us to identify 24 proteins and a pseudogene (ΨBAB1_1059) containing REC domains, the receiver-domain signature for RRs (S3 Table). One of these REC-containing proteins is a hybrid HK (BAB1_0346), three only contain a REC domain (BAB1_0099, BAB2_0628 and BAB2_0042), 18 are linked to a DNA binding domain and the remaining two are linked to a GGDEEF (BAB2_0630) and a sigma-like (BAB1_1671) domain (S3 Table). Based on the fact that the REC domain determines the specificity of interaction with a HK, we cloned all REC domains from the containing genes from *B*. *abortus* (excluding the hybrid HK) into a bacterial two-hybrid vector, and screened this collection using full-length *Brucella* LOVHK as bait. A positive control was included to test the functionality of this two-hybrid experiment. The PdhS HK gene which is known to interact with DivK [35] was cloned into the same bait vector and used to screen the REC minilibrary (Table 1).

**Table 1:** Screening of a minilibrary of *Brucella* REC proteins.

Prey	Bait	Input clones^1^	Positive clones^2^	Double positive clones^3^	Analyzed clones^4^
REC minilibrary	LOVHK	39,800 (100)	>500 (1.3)	>500	20 (100% LovR)
	PdhS-HK	15,600 (100)	>500 (3.2)	>500	5 (100% DivK)
	Empty vector	23,500 (100)	23 (0.1)	ND	ND
REC minilibrary (without LovR)	LOVHK	57,000 (100)	>300 (0.52)	>60	10 (70% PhyR)
	Empty vector	51,000 (100)	139 (0.27)	ND	ND

^1^ Input clones were calculated from the number of colonies that grew in non-selective screening medium (see Materials and Methods for details).

^2^ Number of positive clones that grew in selective screening medium. The percentage of positive clones relative to total clones is indicated between parentheses.

^3^ Number of positive clones that grew in dual selective screening medium.

^4^ Number of positive clones analyzed by PCR and sequencing. RR identity and percentage is indicated between parentheses. ND: not determined.

**Fig 1:**
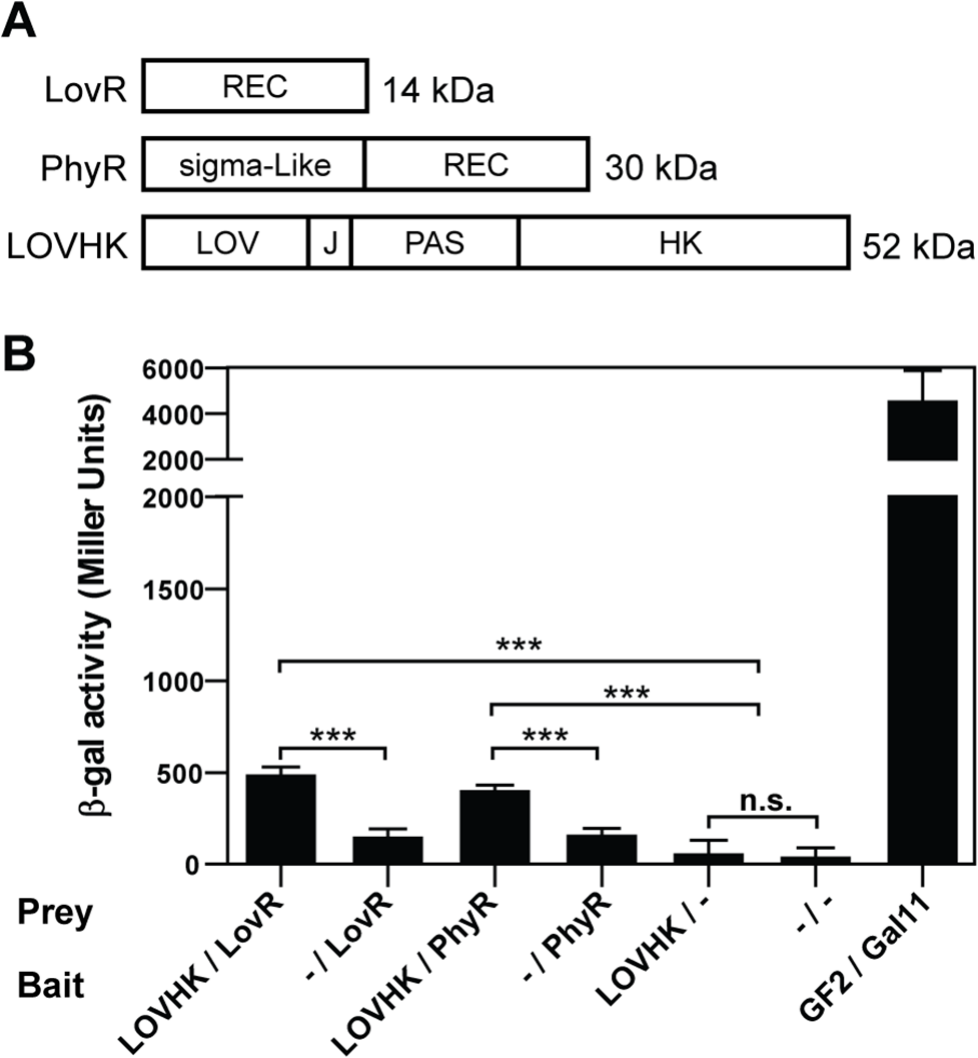
Quantification of the interactions between LOVHK/LovR and LOVHK/PhyR. A. Schematic representation of the LovR, PhyR and LOVHK full-length proteins. The domain composition of each protein is indicated in the boxes. J corresponds to J-alpha-helix present in the C-terminal of LOV domains [49]. B. Equal amounts of each pBT and pTRG plasmids containing the indicated genes were co-transformed in the *E*. *coli* FW 102 O_L_2-62 reporter bacterial strain. Protein interactions were quantified by β-galactosidase activity. Interactions with the corresponding empty vectors (-) were used as negative controls, and the interaction in strains co-transformed with pBT-GF2 and pTRG-Gal11 plasmids was used as a positive control. Data represent the mean ± standard deviation of two independent experiments. *p*-values were determined by one-way ANOVA, followed by Bonferroni’s multiple comparisons test (*** = p<0.001).

Both baits, LOVHK and PdhS, gave a similar number of positive clones, while with the empty vector very few colonies were obtained (Table 1). Sequence analysis of five PdhS-double positive clones showed the expected DivK and 20 of the LOVHK-double positive clones showed that all corresponded to the BAB1_0099 gene from *B*. *abortus*. This gene encodes a small RR protein with only a REC domain (Fig 1A). BAB1_0099 was named LovR as it is a single-domain RR that interacts with a HK protein containing a LOV domain, as in the case of LovR and EL_LovR from *C*. *crescentus* [16] and *E*. *litoralis* [15] respectively. However, there is low sequence similarity among them (S1A Fig and S4 Table). In a second round, the BAB1_0099-containing plasmid was removed from the library and the screening using LOVHK as bait was repeated. The total number of positive clones was lower than the first round but higher than control (Table 1). Ten colonies from the double positive clones were analyzed by PCR and sequencing: seven of them coded for the BAB1_1671 gene, two for BAB1_1538 and one for BAB2_0041. Previous results indicate that BAB1_1538 and BAB2_0041 interact with other HK proteins (S3 Table) [35]. The RR encoded by BAB1_1671 corresponds to PhyR, and it has been previously identified as part of the General Stress Response (GSR) system in *B*. *abortus* [31]. We centered our study in the most relevant interactions observed: LOVHK with LovR, and LOVHK with PhyR. In order to test for the observed interactions between both RRs and LOVHK, equal amounts of each clone were co-transformed with the LOVHK bait vector into the FW 102 O_L_2-62 *E*. *coli* reporter strain [37], which allows quantification of the interaction between the bait and prey by induction of β-galactosidase protein expression. Both RRs showed a significantly higher interaction with LOVHK than with the empty vectors used as negative controls (Fig 1B). These results suggest that LOVHK interacts with two RRs: LovR and PhyR.

### Phosphotransfer from LOVHK to LovR and PhyR

**Fig 2:**
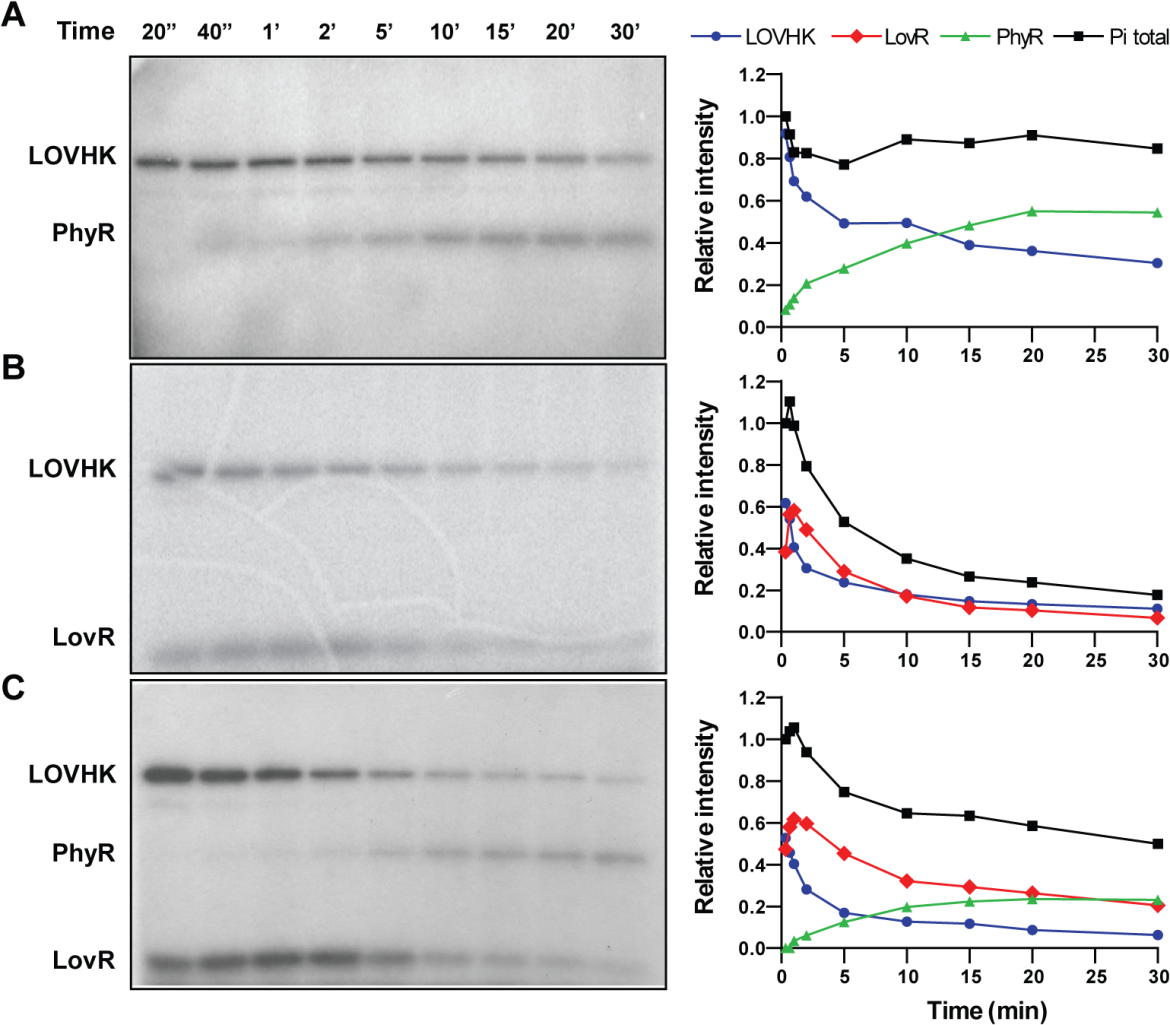
Phosphotransfer reaction between *Brucella* LOVHK and RRs. Purified LOVHK protein was illuminated in phosphorylation buffer containing [γ-^32^P] ATP. After 15 min at 37°C, purified response regulators were added to the mixture to a final concentration of 2.5 μM each for the three proteins. The final concentration of LOVHK was also 2.5 μM. At the indicated times after addition of the corresponding response regulators, aliquots were drawn and separated by 15% SDS-PAGE. Autoradiograms are shown on the left, and the graphs on the right side indicate the relative intensity of each band to the total intensity at time 20 seconds. The experiment was repeated three times, and a representative experiment is shown. Numbers above the autoradiograms indicate the time in seconds (columns 1 and 2) or in minutes (columns from 3 to 9) respectively. A. Phosphotransfer between LOVHK and PhyR. B. Phosphotransfer between LOVHK and LovR. C. Phosphotransfer between LOVHK, PhyR and LovR simultaneously. LOVHK: blue circles, PhyR: green triangles, LovR: red rhomboids, total intensity: black squares. Molecular weights of protein constructions are indicated in Fig 1A.

RRs are activated by the transfer of a phosphoryl group from a histidine residue in the HK domain to a conserved aspartic acid residue in the REC domain of the RR [6]. Both LovR and PhyR contain the conserved aspartic acid that is the putative phosphor-acceptor residue (highlighted in yellow in S1B Fig). In order to determine whether PhyR and LovR could be phosphorylated *in vitro* by LOVHK, we first conducted a time course for LOVHK autophosphorylation. After a brief white-light pulse, LOVHK phosphorylation reached saturation after 15–30 min in subsequent darkness (S2 Fig). The unirradiated control showed a similar time course although the saturation level was lower than that of the illuminated sample. There followed a slow loss of phosphate for the illuminated sample over the next several hours. Then, we conducted phosphotransfer assays, by adding recombinant purified PhyR or LovR to a previously autophosphorylated LOVHK. Firstly, recombinant LOVHK was preloaded with phosphate by briefly irradiating it with white light in the presence of radioactive [γ-^32^P]-ATP and incubating for 15 min prior to the addition of each RR. The 15 min wait insured strong LOVHK labeling at the time of RR addition (S2 Fig). Both RRs were phosphorylated by LOVHK, although they presented different kinetics (Fig 2A and 2B). PhyR phosphorylation becomes evident after 40 seconds of incubation and reaches a plateau after 20 minutes, coinciding with a drop in the phosphorylation level of LOVHK (Fig 2A). LovR shows a fast increase in phosphorylation level reaching a maximum after 2 minutes and then becomes dephosphorylated in 10 minutes (Fig 2B). When LOVHK is incubated with PhyR, the total amount of phosphate of the system remains constant suggesting that the phosphorylation level of this RR is stable (Fig 2A). However, the addition of LovR strongly decreases the phosphorylation level of LOVHK and consequently the total amount of phosphate present in the system (Fig 2B). This result suggests that the phosphate transferred to LovR is rapidly hydrolyzed. In order to test whether LOVHK is able to phosphotransfer to PhyR in the presence of LovR, we repeated the autophosphorylation labeling of LOVHK and added stoichiometric amounts of both RRs at the same time (Fig 2C). The phosphorylation kinetics of LovR was similar to the pattern observed in the previous experiment where the RR was incubated with LOVHK (Fig 2B). The PhyR protein was also phosphorylated although the kinetics was slightly slow as compared when this RR was alone with LOVHK (Fig 2A), which led to a minor level of PhyR~P (Fig 2C). However, the presence of LovR did not completely inhibit the phosphotransfer to PhyR indicating that LOVHK is able to interact with both RRs simultaneously. Under similar conditions, the phosphorylated HK domain of LOVHK did not phosphotransfer to an unrelated *Brucella* RR, NtrX (S3 Fig), and a similar result was obtained when PhyR was incubated with an unrelated *Brucella* HK, PAS-HK-NtrY (S4 Fig). Taking together, these results strongly suggest that the interaction between LOVHK with both RRs is specific and that LOVHK is able to phosphotransfer to the central regulator of the GSR system in *Brucella*.

### LOVHK modulates the GSR response

PhyR is one of the main components of the GSR system in α-proteobacteria, which has recently been described in *B*. *abortus* 2308 [31] and other microorganisms belonging to the same group [17–21, 24, 25, 27–30]. The GSR system positively regulates its own transcription, so that activation of the GSR system by a stress factor leads to an increase in the expression of genes belonging to the GSR system including *phyR*, *nepR* and *rpoE*. Stationary phase is generally associated with nutrient starvation, which is a stress factor and leads to the activation of the GSR system in many Gram-positive and Gram-negative bacteria [20, 25, 26, 50]. Therefore, bacteria in stationary phase are more resistant to changes in the environmental conditions compared to exponential phase. As a consequence, all experiments were conducted with bacteria under exponential phase, in order to avoid the stress-induction effect in stationary phase.

**Fig 3:**
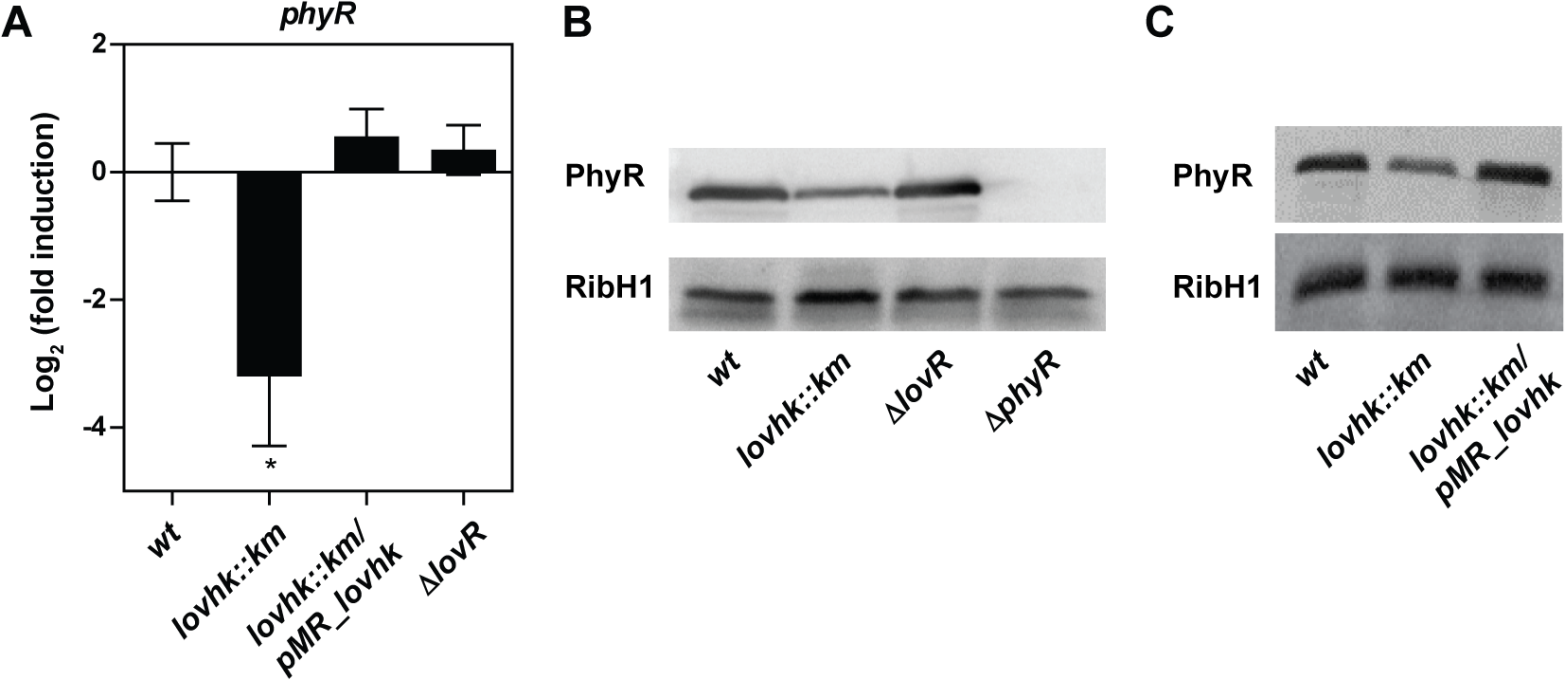
PhyR expression is decreased in the *lovhk* mutant. A. *B*. *abortus* 2308 wt and the isogenic *lovhk*::*km*, *∆lovR* and *lovhk*::*km*/pMR_*lovhk* strains were cultured in TSB up to logarithmic phase, and expression of the *phyR* gene was analyzed by qRT-PCR. The *if-1* housekeeping gene was used as a reference. Data represent the average of three independent experiments, and are reported as fold induction relative to wt ± standard error. *p*-values between each strain and wt were determined by one-way ANOVA and post-hoc Tukey’s multiple comparisons test (* = p<0.05). B. The PhyR protein levels were analyzed by western blot in *B*. *abortus* 2308 wt and in the isogenic *lovhk*::*km*, *∆lovR* and *∆phyR* mutant strains using a mouse polyclonal antibody anti-PhyR. Bacteria were grown in TSB and processed in logarithmic phase. The experiment was repeated three times with similar results. A representative experiment is shown. C. The PhyR protein levels were analyzed by western blot in *B*. *abortus* 2308 wt and in the isogenic *lovhk*::*km* and *lovhk*::*km/*pMR_*lovhk* strains. Bacteria were grown in TSB and processed in logarithmic phase. The experiment was repeated three times with similar results. A representative experiment is shown. Polyclonal anti-RibH1 was used as loading control. PhyR: 30 kDa; RibH1: 16.8 kDa.

In order to test whether LOVHK can modulate the GSR system in the absence of any stress, we estimated the basal activation of the GSR system by following *phyR* mRNA expression. Wild type and the isogenic *lovhk*::*km* and *∆lovR* mutant strains were grown in rich medium up to logarithmic phase and the amount of *phyR* expression was measured by qRT-PCR. We found that *phyR* expression was significantly decreased in the *lovhk*::*km* mutant compared to wt, while the *∆lovR* mutant did not show any difference (Fig 3A). In order to verify if this effect was due to the absence of *lovhk*, we introduced a plasmid expressing this gene under the control of its own promoter (pMR10*cat*_*lovhk*) in the *lovhk*::*km* mutant strain. The *phyR* expression of this complemented strain (named *lovhk*::*km*/pMR_*lovhk*) was similar to wt and significantly different from the *lovhk*::*km* (Fig 3A). In addition, we analyzed PhyR protein levels under the same conditions by western blot to assess whether the protein level was also modified in the mutant strains. As expected, we found that the *∆phyR* mutant strain did not express PhyR and that PhyR protein level was decreased in the *lovhk*::*km* mutant as compared to wt. However, it did not show a significant change in the *∆lovR* mutant strain (Fig 3B). In the case of the *lovhk* complemented strain, it showed a PhyR protein level similar to the wt strain (Fig 3C). Taking together, these results suggest that LOVHK is required for maintaining the basal expression level of PhyR in rich media.

Furthermore, as LOVHK is a light sensor, we tested whether *phyR* expression varied between light and dark conditions in the wt strain. However, we did not find a difference in RNA and protein expression of PhyR (data not shown), even though we tried different periods of time of exposure to light and culture media.

**Fig 4:**
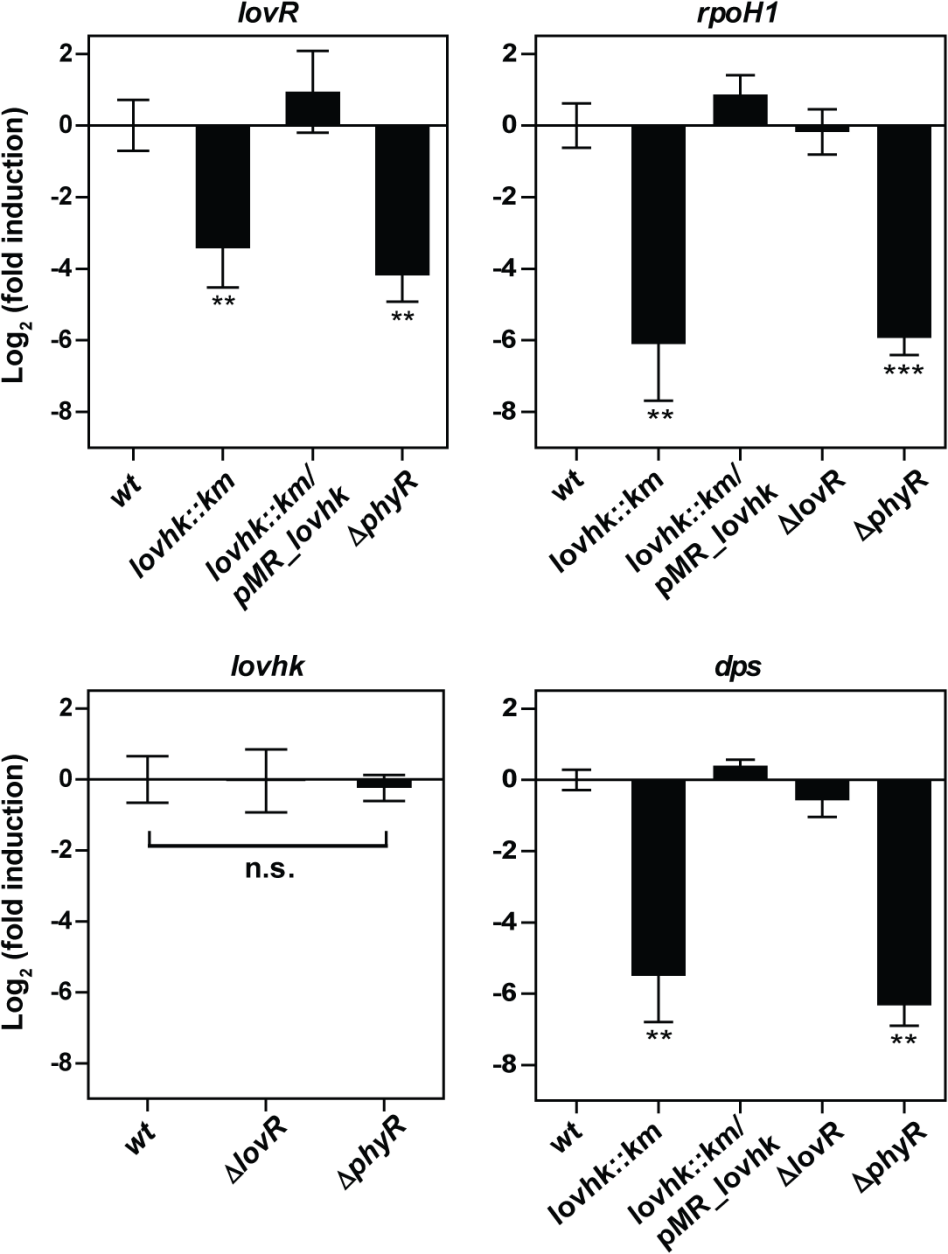
LOVHK modulates the GSR system in *B*. *abortus*. *B*. *abortus* 2308 wt and in the isogenic *lovhk*::*km*, *∆lovR*, *∆phyR* and *lovhk*::*km*/pMR_*lovhk* strains were cultured in TSB up to logarithmic phase, and expression of *lovhk*, *lovR*, *dps* and *rpoH1* genes was analyzed by qRT-PCR. The *if-1* housekeeping gene was used as a reference. Data represent the average of three independent experiments, and are reported as fold induction relative to wt ± standard error. *p*-values between each strain and wt were determined by one-way ANOVA and post-hoc Tukey’s multiple comparisons test (** = p<0.01; *** = p<0.001).

Kim *et al*., 2013, identified a series of genes that are regulated by the GSR system in *B*. *abortus*, including *rpoH1* (RNA polymerase factor sigma-32 RpoH1), *dps* (DNA starvation/stationary phase protection protein Dps) and *lovR*. Thus, we decided to investigate further whether the expression of these genes is also modified in the absence of LOVHK. The *B*. *abortus* 2308 wt and the isogenic *lovhk*::*km*, *∆lovR*, *∆phyR* and *lovhk*::*km*/pMR_*lovhk* strains were grown in rich medium up to logarithmic phase. After RNA extraction, the expression of *rpoH1*, *dps* and *lovR* genes was measured by qRT-PCR. We found that expression of these three genes was significantly down-regulated in both the *lovhk*::*km* and *∆phyR* mutant strains compared to wt, and fully restored in the *lovhk*::*km*/pMR_*lovhk* complemented strain (Fig 4). By contrast, *rpoH1* and *dps* gene expression was not modified in the *∆lovR* mutant. Moreover, *lovhk* basal expression was not modified in the *∆lovR* and *∆phyR* mutant strains compared to wt (Fig 4). These results suggest that LOVHK contributes to regulate the basal transcription level of genes regulated by the GSR system in *B*. *abortus in vivo*.

**Fig 5:**
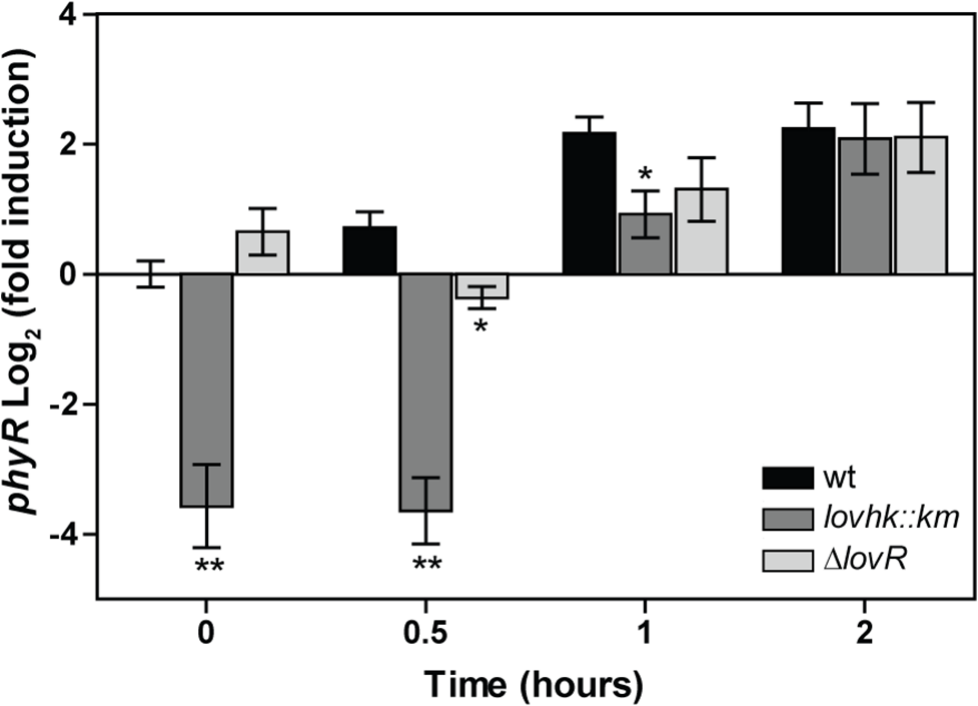
Induction of the GSR system under starvation. *B*. *abortus* 2308 wt (black bars) and the isogenic *lovhk*::*km* (dark grey bars) and *∆lovR* (light grey bars) mutant strains were grown in TSB medium up to logarithmic phase. First, an aliquot was drawn (time 0 h), then the rest of the culture was washed and resuspended in modified MM1 minimal medium, and aliquots were drawn at 0.5 h, 1 h and 2 h. Expression of *phyR* gene was analyzed by qRT-PCR. The *if-1* housekeeping gene was used as a reference. Data represent the average of three independent experiments and are reported as fold induction relative to wt at time 0 h in TSB ± standard error. *p*-values between each strain and wt (at each time point) were determined by one-way ANOVA and post-hoc Tukey’s multiple comparisons test (* = p<0.05; ** = p<0.01).

In many α-proteobacteria, the GSR system responds to different stress factors including exposure to hydrogen peroxide, UV light, high osmolarity, nutrient starvation, or desiccation [17, 18, 20, 21, 24, 25, 27]. We tested whether starvation could induce the GSR system. *Brucella* wt cells were grown up to logarithmic phase, transferred to a modified MM1 minimal medium and *phyR* expression was determined. After 2 h in this medium, bacterial viability was not affected (S5 Fig) and the GSR system was induced indicating that starvation condition can trigger the stress response (S6 Fig). Thus, in order to test if the GSR system is activated by starvation in the mutants, *Brucella* cells were grown in rich medium up to logarithmic phase and transferred to a modified MM1 minimal medium, and *phyR* epression was analyzed at different time points (Fig 5). During the time of the experiment, bacterial viability was not affected (S5 Fig). The *lovhk*::*km* mutant strain showed diminished expression at time 0 h as compared with wt strain. Starvation condition was able to induce the expression level of *phyR* in the three strains evaluated. However, the induction profile of *lovhk*::*km* mutant was delayed with respect to wt control. After 1 h of incubation the wt reached the fully induction state while in the *lovhk*::*km* mutant the induction level was still reduced. At 2 h, the *phyR* expression level was similar in the three strains. The *∆lovR* mutant strain showed a *phyR* induction profile not significantly different from wt, except at 0.5 h that showed a slight decrease (Fig 5). In conclusion, the absence of LOVHK does not prevent the GSR from responding to starvation, but LOVHK is needed to obtain the fully induction state in a short period of time under starvation stress.

**Fig 6:**
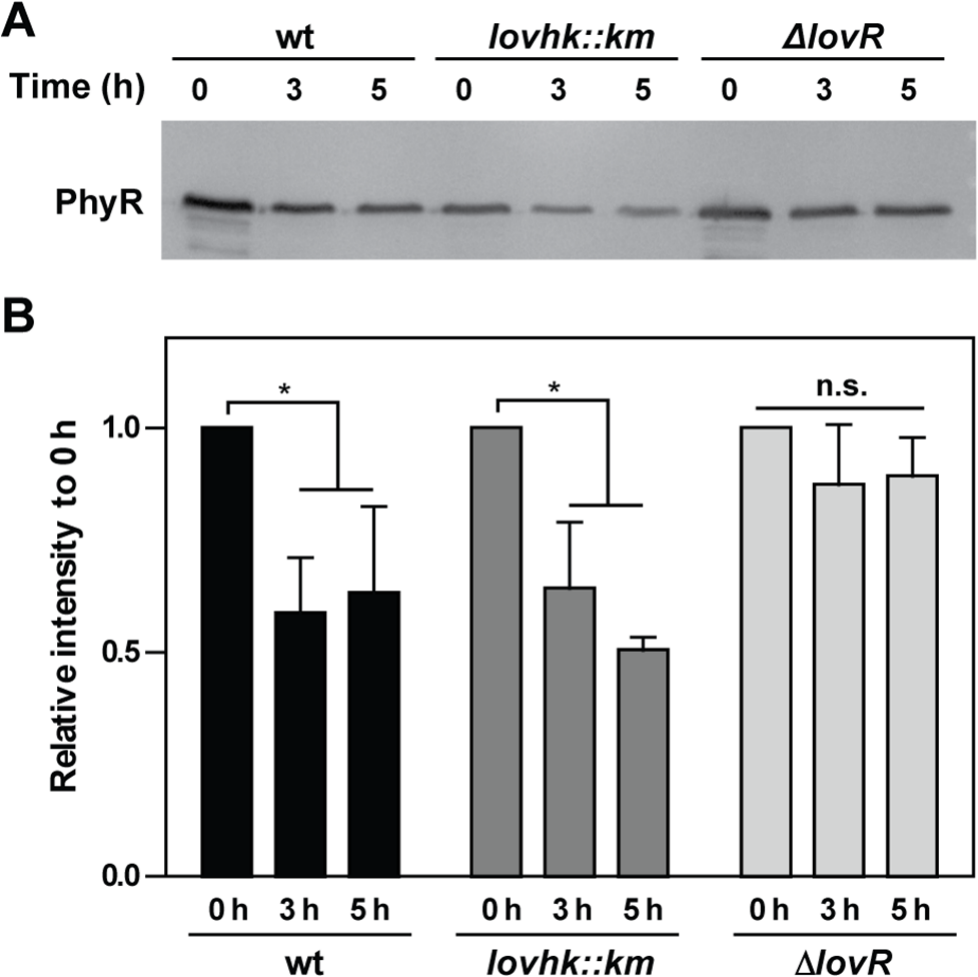
PhyR stability. *B*. *abortus* 2308 wt (black bars) and the isogenic *lovhk*::*km* (dark grey bars) and *∆lovR* (light grey bars) mutant strains were grown in TSB medium up to logarithmic phase. First, an aliquot was drawn (time 0 h), and then chloramphenicol was added to the rest of the culture in order to inhibit protein synthesis. Samples were taken after 3 h and 5 h. The experiment was repeated three times **A.** The PhyR protein abundance was analyzed by western blot, and a representative experiment is shown. **B.** The PhyR protein levels of the three experiments were quantified. Columns indicate the average of relative intensity to time 0 h ± standard deviation for each strain. *p*-values were determined by one-way ANOVA for repeated measures and a post-hoc paired t-test (* = p<0.05). PhyR: 30 kDa.

Kim *et al*., 2013, reported that PhyR protein levels decrease in the absence of stress by the action of the ClpXP protease. However, in the presence of stress PhyR protein levels are stabilized by phosphorylation and/or inactivation of ClpXP. Therefore, we decided to analyze whether LOVHK and LovR affect PhyR protein stability *in vivo*. *B*. *abortus* wt, *lovhk*::*km* and *∆lovR* mutant strains were grown in rich medium up to logarithmic phase and then the protein synthesis was inhibited by adding chloramphenicol. In the wt and *lovhk*::*km* mutant strains, the PhyR protein level decreased during the experiment with a similar profile (Fig 6). By contrast, in the *∆lovR* mutant strain PhyR protein level did not significantly changed during the 5 hours of the experiment. Although the effect seems to be modest, this result suggests that PhyR is more stabilized in the absence of LovR.

### Deletion of LOVHK affects the *virB* operon expression

Swartz *et al*., 2007, found that the *lovhk*::*km* mutant is less infective than the wt strain in the J774A.1 macrophage cell line. However, neither *∆lovR* nor *∆phyR B*. *abortus* mutants showed significant differences compared to wt in cell infection assays (data not shown). Kim *et al*., 2013, also reported a similar result for the *B*. *abortus ∆phyR* and *∆rpoE1* strains in primary murine macrophage infection and initial colonization of BALB/c mouse spleens. However, they found that the GSR system is important for maintenance of a chronic infection in mice (more than 1 month p.i.). In addition, *B*. *melitensis* 16M *ΔrpoE1* mutant strain becomes attenuated in BALB/c mice at 4 weeks p.i. [51]. While the GSR system in *Brucella* is important for persistence in mice and not at short times p.i., LOVHK is important during the early infection phases. Therefore, apart from the GSR system, LOVHK may be signaling to other intracellular components. Although there have been described many virulence factors in *Brucella*, the most characterized is the *virB* operon that encodes a Type IV Secretion System (T4SS). This secretion system translocates protein effectors from the bacterium to the cytoplasm of the eukaryotic cell, modifying the response of the host [52]. Interruption of *virB* genes leads to complete loss of virulence both in cell cultures and in mice [53]. Thus, v*irB* expression is crucial for macrophage infection, survival and establishment of the replicative niche inside host cells.

**Fig 7:**
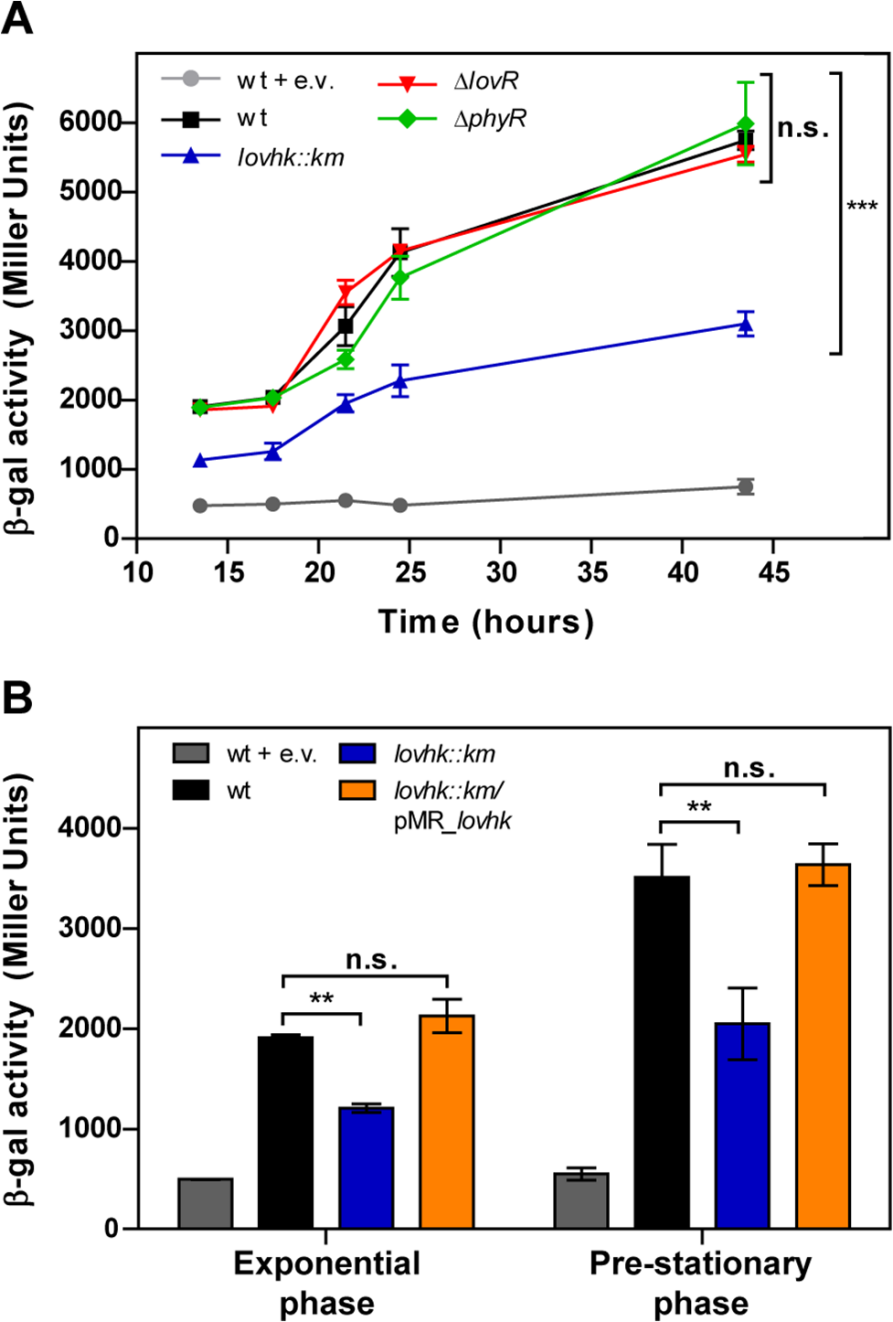
The *virB* promoter activity is decreased in the *lovhk*::*km* mutant in *B*. *abortus*. A. The *virB* promoter activity was assessed using a transcriptional fusion of the *virB* promoter to *lacZ*. *B*. *abortus* 2308 wt and the isogenic *lovhk*::*km*, *∆lovR* and *∆phyR* mutant strains were transformed with pBBR-prom-*virB*-*lacZ* replicative vector. Bacteria were cultured in TSB until stationary phase, and β-galactosidase activity was assessed at different time points. Color code: *B*. *abortus* 2308 wt (black), *lovhk*::*km* (blue), *∆lovR* (red), *∆phyR* (green), wt + e.v. (grey) and *lovhk*::*km*/pMR_*lovhk* (orange). B. The *B*.*abortus* 2308 wt, *lovhk*::*km* and *lovhk*::*km*/pMR_*lovhk* strains transformed with the pBBR-prom-*virB-lacz* vector were cultured in TSB and β-galactosidase activity was assessed at exponential and pre-stationary phases. In both experiments the wt strain transformed with pBBR-*lacZ* empty vector was used as a negative control (wt + e.v.). Data of both panels are reported in Miller Units as mean ± standard deviation of two biological samples from one representative experiment. Both experiments were repeated three times with similar results. *p*-values between each strain and wt were determined by one-way ANOVA and post-hoc Tukey’s multiple comparisons test (** = p<0.01, *** = p<0.001). Color code: *B*. *abortus* 2308 wt (black), *lovhk*::*km* (blue), wt + e.v. (grey) and *lovhk*::*km*/pMR_*lovhk* (orange).

When bacteria are grown in rich medium, *virB* is transcriptionally activated at the beginning of the stationary phase [53]. Hence, we tested whether LOVHK could modify *virB* expression. For this purpose, we measured the activity of the *virB* promoter by a transcriptional fusion to the *lacZ* reporter gene in *B*. *abortus* 2308 wt and in *lovhk*::*km*, *∆lovR* and *∆phyR* mutant strains. Bacteria were grown in rich medium and β-galactosidase activity was assayed at different times from exponential to stationary phases (S7 Fig). The activity of the *virB* promoter is induced when *Brucella* enters in the stationary phase in all strains. However, the induction of *virB* promoter showed a decrease of about 50% in the *lovhk*::*km* mutant as compared to the wt. While in *∆lovR* and *∆phyR* strains the activity of the *virB* promoter was very similar to wt (Fig 7A). Complementation of *lovhk*::*km* with the plasmid expressing LOVHK protein completely restores the *virB* induction (Fig 7B). This result suggests that deletion of *lovhk* decreases induction of the *virB* promoter by a GSR independent mechanism.

## Discussion

In a previous study, LOVHK was described as a blue-light sensor *in vitro*, as exposure to light increases its autophosphorylation activity. It was also found to be a virulence factor, as a *lovhk*::*km* mutant strain presents an attenuated phenotype in cell culture infections [10]. Herein, we have characterized a novel and functional signaling pathway in *Brucella* where three components have been elucidated, LOVHK and two RRs: LovR and PhyR. The results presented in this work allow us to propose that *Brucella* LOVHK is signaling to the GSR system and affects the expression of the *virB* operon, an important factor in bacterial virulence.

In *B*. *abortus* both *lovhk* (BAB2_0652) and *lovR* (BAB1_0099) are coded in different chromosomes, with no apparent partner in their vicinity. In *C*. *crescentus*, *lovK* and *lovR* are encoded in a single locus [16]. The genome of *E*. *litoralis* encodes three *lov-hk* genes. *EL362_lovhk* and *lovR* are encoded in an operon, while *EL368_lovhk* and *EL346_lovhk* are orphan HKs, with no predictable cognate RR in the proximate region [15]. Taking into account the genomic localization of these LOV-HKs and the single-domain RRs that interact with them, this information suggests that LOV-HKs cognate partners are not easily predictable, and that their identification needs to be studied carefully.

Using bacterial two-hybrid assays and phosphotransfer experiments (Table 1, Figs 1 and 2), we identified two RRs as partners of LOVHK. Activation of LOVHK leads to an increase in autophosphorylation activity and phosphotransfer to its two RRs partners. Phosphotransfer from LOVHK to LovR presents a faster kinetics than transfer of phosphoryl moiety to PhyR. However, the phosphoryl group attached to PhyR~P is more stable than the LovR~P, which loses its phosphoryl group in a few minutes and leads to a rapid loss of LOVHK phosphate. Based on the *in vitro* phosphotransfer assays, we propose that LovR could be functioning as a phosphate-sink resulting in LOVHK inactivation *in vitro*. The LovR dephosphorylation could be due to self-catalyzed dephosphorylation or by a possible phosphatase activity of LOVHK [54, 55]. The mechanism of LovR dephosphorylation will be characterized in a further work.

Previous works described analogous signaling systems also composed of a LOV-domain-containing HK belonging to the HWE family, a single-domain RR and PhyR in *C*. *crescentus* and *E*. *litoralis*. In *C*. *crescentus*, the relationship between LovK and both RRs, LovR and PhyR, was demonstrated by a genetic approach [14, 16], while in *E*. *litoralis* the interactions among these proteins was studied by *in vitro* phosphotransfer assays [15]. In *E*. *litoralis* the three LOV-HK proteins, named EL368_LOVHK, EL346_LOVHK and EL362_LOVHK differ in their phosphotransfer profiles. While EL368_LOVHK and EL346_LOVHK phosphorylate both RRs *in vitro*, EL362_LOVHK only transfers phosphate to LovR. The phosphorylation kinetics from *Brucella* LOVHK to PhyR is very similar to the kinetics from EL368_LOVHK to PhyR. In both cases, PhyR~P signal intensity stabilizes after 20–30 minutes of incubation. However, the dephosphorylation kinetics of LovR~P is faster in *Brucella* than in *E*. *litoralis*. We have not observed an increase in the dephosphorylation rate of PhyR~P when LovR is present in the reaction (Fig 2C) such as was found in *E*. *litoralis* PhyR [15]. Two possible scenarios could explain this observation: A) PhyR is dephosphorylated by LovR and LOVHK re-phosphorylates again PhyR protein maintaining the level of PhyR label; B) LovR only acts as a phosphate-sink for LOVHK and the phosphorylated PhyR is stable. Further work is needed to elucidate between these two alternative hypotheses.

In *B*. *abortus*, in the absence of stress, LOVHK increases basal transcription of *phyR* and other genes regulated by the GSR system (Figs 3 and 4). Upon stress by starvation, the absence of LOVHK does not impair the final GSR response, but LOVHK is needed to obtain the fully induction state in a shorter period of time (Fig 5) thus, confirming that LOVHK contributes to the activation of the GSR system. On the other hand, even though LovR decreases the phosphorylation state of LOVHK *in vitro*, contributing to return LOVHK to the inactive state, it does not have a significant impact on transcription of the GSR regulon *in vivo* (Figs 2C, 3 and 4). The effect of mutating the *lovR* gene could be compensated by other not yet characterized mechanisms. However, the absence of LovR increases PhyR stability *in vivo* (Fig 6). This effect could be the consequence of a decreased level of LOVHK~P when LovR is present, a condition that finally leads to lower phosphorylation levels of both RRs. Further work is needed to characterize in more detail the *in vivo* role of LovR.

The expression of *lovhk* is not regulated by the GSR system in *B*. *abortus* under normal growing conditions, while *lovR* transcription depends on GSR activation (Fig 4). These results are in agreement with a previous microarray analysis showing that *lovhk* expression is not modified in the *B*. *abortus rpoE1* mutant as compared with the wt strain under oxidative stress conditions, while *lovR* expression is markedly decreased in the same conditions [31]. Additionally, neither the *lovhk* nor the *lovR* promoters have the DNA sequence motif recognized by RpoE1, suggesting that *lovR* is only indirectly regulated by the GSR system. On the contrary, in *C*. *crescentus* the σ^T^ (the orthologue of RpoE1) recognition motif is present in the promoter of *lovK* and *lovR* genes, and their transcription is regulated by the GSR system showing that both genes are directly regulated by the sigma factor σ^T^ [14].

The GSR system is responsive to many environmental conditions suggesting that PhyR integrates the input signals of several sensory HKs. In α-proteobacteria, the GSR system is characterized for a conserved genomic context, which involves the genes coding for the ECF sigma factor *rpoE*, *nepR* and *phyR*, and in many cases also includes one or two sensor HKs [17, 56]. Three of this kind of sensor HKs have been characterized as possible regulators of PhyR phosphorylation level: PhyK from *C*. *crescentus* [21], PhyP from *Sphingomonas* sp. Fr1 [24], and RsiC from *S*. *meliloti* [57]. In *Brucella*, the GSR genomic locus is also associated with two genes coding for putative HKs: BAB1_1669 and BAB1_1673. At the time of writing, both HKs have been analyzed and none of them phosphorylate PhyR [58]. However, our results suggest that in *Brucella* other HKs could be activating PhyR, as the absence of LOVHK does not impair the GSR system to respond to stress under starvation conditions (Fig 5).

*Brucella* has many virulence factors, although the most characterized is the T4SS *virB* system, which is essential for *Brucella* infection [52]. The regulation of the *virB* operon is very complex, involving at least five regulatory factors [59]. Herein, we show that the absence of LOVHK markedly decreases the *virB* operon promoter activity by a GSR independent manner (Fig 7). This result suggests that, in addition to modulation of the GSR system, LOVHK may be participating in other signaling pathways. Evidence in favor of this hypothesis is that *virB* expression was not modified in a microarray analysis conducted between *B*. *abortus* wt and the *∆rpoE1* mutant strain under stress conditions induced by hydrogen peroxide [31]. Moreover, the consensus RpoE1 recognition motif [17, 31, 56] was not found in the *virB* promoter from *B*. *abortus* and *B*. *melitensis* [60]. These results suggest that the *virB* operon is not directly regulated by the GSR system. However, a decreased amount of VirB8 protein expression was reported in a *B*. *melitensis ∆rpoE1* mutant strain grown under normal conditions [51]. Altogether, these results suggest that LOVHK may be controlling gene expression by at least two signaling pathways, and the mechanism by which *virB* expression is altered in the absence of *lovhk* remains unknown.

At the time of writing, Gourley *et al*., 2014, demonstrated by a microarray analysis that genes related to the GSR system and *virB* operon are differently regulated in a *lovhk* mutant strain of *B*. *melitensis* as compared with wt [61]. Here we further demonstrated a biochemical and physiological regulation of these genes in *B*. *abortus*, characterized the LOVHK signaling pathway and identified new components of the GSR system.

In addition to other stress factors, light is also considered a stress signal. Even though it has been demonstrated that *Brucella* LOVHK is a blue-light sensor protein *in vitro* and that light modulates bacterial virulence in macrophage cell line infections, we could not demonstrate light as a modulator of the GSR system in bacterial cultures. However, the presence or absence of LOVHK modulates the GSR response. A similar result was obtained for *C*. *crescentus* LovK, and the authors suggested that LovK could be sensing the cytoplasmic redox potential through the LOV domain [14, 62]. Further experiments will test whether *Brucella* LOVHK is sensing another signal besides blue-light. Recent studies of Kim *et al*., 2014 suggest that the PAS domain of *Brucella* LOVHK is involved in the response to oxidative stress [58].

*Brucella* can be acquired by aerosols, direct contact with mucosa or skin wounds with body fluids from infected animals, or by consumption of contaminated dairy products. Light sensed by bacteria present in aborted placenta may prepare the bacteria for infection of the following host [10]. Kim *et al*., 2013, demonstrated that *B*. *abortus* PhyR~P/NepR is a long-lived complex, so that when PhyR is activated by phosphorylation, bacteria would be prepared not only for the present stress factor, but also for future stresses, as has also been proposed for *M*. *extorquens* [18], and *B*. *japonicum* [20]. In accordance with this model, light or some other signal sensed by LOVHK prior to infection may prepare *Brucella* for future stresses encountered in the host. However, in the absence of PhyR or RpoE1, alternative mechanisms may compensate in order to respond to stresses encountered in the host.

**Fig 8:**
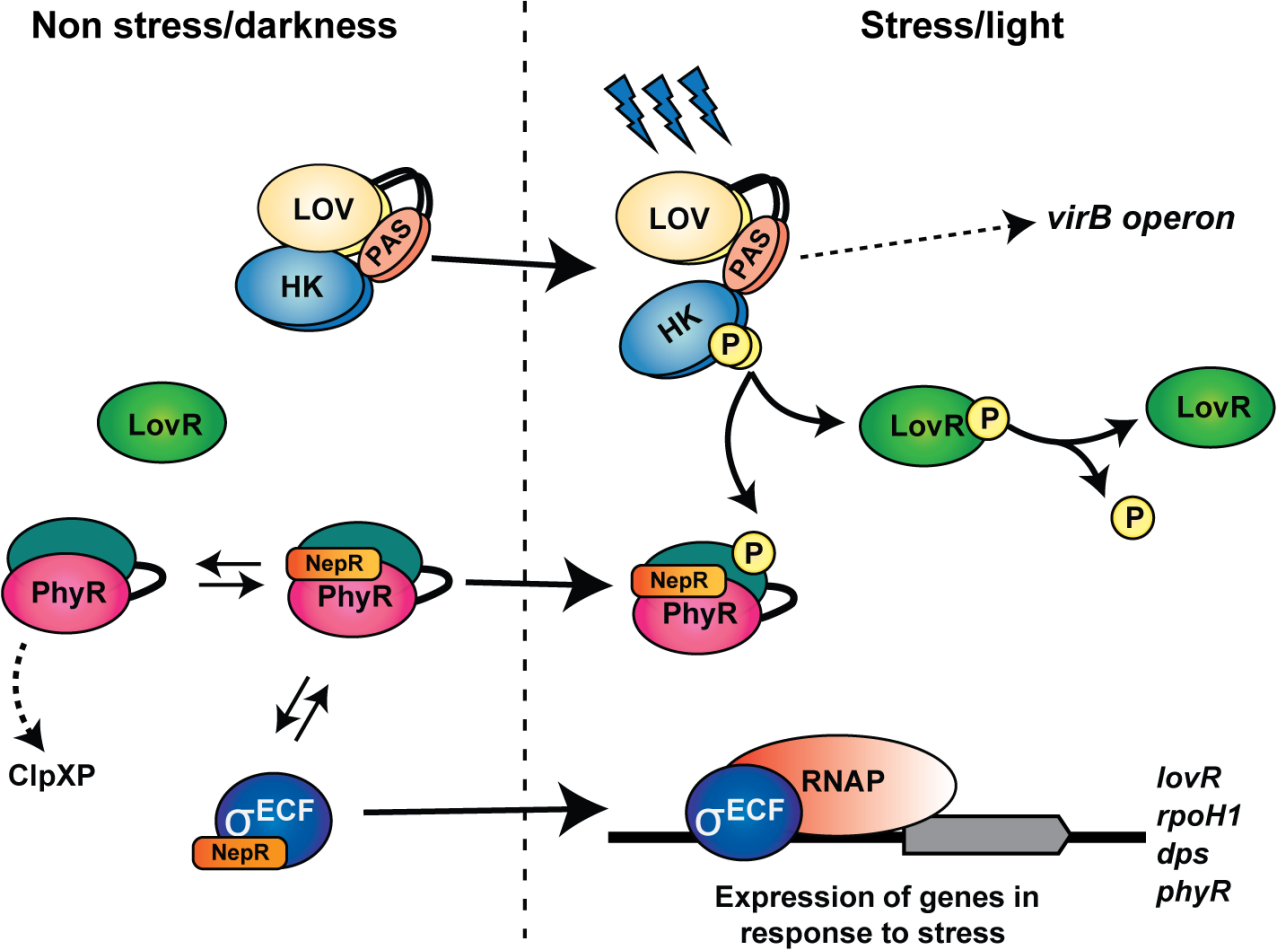
Proposed model for the LOVHK-GSR regulatory pathway. In the absence of stress or under dark conditions, LOVHK is inactive and NepR can interact with both unphosphorylated PhyR and RpoE1 (σ^ECF^), thus preventing RpoE1 association with RNAP (RNA polymerase). Upon blue-light exposure or other stress signal, LOVHK is activated, it autophosphorylates and, subsequently, transfers the phosphate moiety to LovR and PhyR. PhyR phosphorylation stabilizes PhyR~P/NepR complexes, thus releasing RpoE1, which can now associate with the RNAP. The RNAP-RpoE1 complex is now able to regulate directly or indirectly the expression of stress-related genes such as *rpoH1*, *dps*, *phyR* and *lovR*. LOVHK, probably indirectly, up-regulates the expression of *virB* operon in a GSR independent manner. Finally, the evidence from *in vitro* experiments suggests that LovR decreases LOVHK phosphorylated state. In summary, LOVHK contributes to the activation of the GSR system and affects the *virB* operon expression by an unknown mechanism (dashed arrow). Arrows with a continuous line suggest a direct interaction.

In conclusion, this report provides important insights into the LOVHK signaling cascade. Our work identifies a novel TCS pathway in *Brucella*, composed of LOVHK, LovR and PhyR, and defines a functional role for the light sensor LOVHK. We also identified the first HK that interacts with PhyR in this bacterium, and we establish a connection between LOVHK, the GSR system and *virB* expression. Combining the results presented here together with the information from previous reports [31, 63], we propose a model for LOVHK intracellular signaling pathway (Fig 8). This model is in concordance with what has already been described for the GSR system in *B*. *abortus*. Furthermore, this is the first mammalian pathogen for which a physiological role and intracellular interactions for a LOV-domain-containing protein have been described.

## Supporting Information

S1 Fig Sequence comparison of *Brucella* LovR and identification of the predicted phosphorylatable aspartic acid of LovR and PhyR. **A.** Alignment of LovR sequences of *Brucella abortus* 2308, *Caulobacter crescentus* CB15 [14], *Erythrobacter litoralis* HTCC2594 [15], and Mext_0407 from *Methylobacterium extorquens* PA1 [64] is shown. **B.** Alignment of *Brucella abortus* LovR and PhyR sequences is shown. In both cases, alignments were carried out with Clustal-Omega2 (EMBL-EBI) [65] and the predicted phosphorylatable aspartic acid is highlighted in yellow. References: ‘*’ fully conserved residue, ‘:’ conservation between groups of strongly similar properties, ‘.’ conservation between groups of weakly similar properties.
(TIF)

S2 Fig Time-course for autophosphorylation of LOVHK in the dark and in the light. Autophosphorylation of LOVHK was carried out in 10 μl of final volume in phosphorylation buffer (20 mM Tris-HCl pH 8, 50 mM KCl and 5 mM MgCl_2_), containing 7.5 μl of purified LOVHK (5 μM) and 1 μCi of [γ-32P] ATP (111TBq/mmol, Perkin Elmer Life Sciences) that had been diluted 10x with cold 10 μM ATP. For the phosphorylation time course, samples were irradiated (light) for one minute of white light (a fluence of 2000 μmol m^-2^ s^-1^ white light; Kodak carousel 4400 projector) or mock-irradiated (dark), and incubation was continued at room temperature under dark conditions. Samples were taken at the indicated times and the reactions were stopped with an equal volume of 2X Laemmli sample buffer prior to SDS-PAGE (15%). The gel was dried and exposed to a phosphor screen (Typhoon) for quantitation. The experiment was done three times with results similar to those shown.
(TIF)

S3 Fig LOVHK does not phosphotransfer NtrX. Purified recombinant HK domain of LOVHK (26 kDa) or PAS-HK-NtrY (42 kDa) at a concentration of 2.5 μM and 10 μM respectively, were incubated in phosphorylation buffer containing [γ-^32^P] ATP (separately). After 15 min at 37°C, the purified recombinant REC domain of NtrX (16 kDa) was added to each reaction mixture to a final ratio Histidine Kinase:NtrX 1:3. Aliquots were drawn at the indicated times and separated by 15% SDS-PAGE, exposed to a Storage Phosphor Screen (GE Healthcare), and scanned by a Storm 840 Molecular Imager (GE Healthcare). An autoradiogram is shown. Phosphotransfer between the HK domain of LOVHK and the REC domain of NtrX is shown on the left, and phosphotransfer between PAS-HK-NtrY and the REC domain of NtrX is shown on the right. Numbers above the autoradiogram indicate the time in seconds (columns 1 and 6) or in minutes (columns from 2 to 5 and columns 7 to 10).
(TIF)

S4 Fig PhyR is not phosphorylated by the unrelated HK, NtrY. Purified recombinant HK domain of LOVHK (26 kDa) or PAS-HK-NtrY (42 kDa) at a concentration of 2.5 μM and 10 μM respectively, were incubated in phosphorylation buffer containing [γ-^32^P] ATP (separately). After 15 min at 37°C, purified recombinant PhyR (30 kDa) was added to each reaction mixture to a final concentration of 2.5 μM. Aliquots were drawn at the indicated times and separated by 15% SDS-PAGE, dried and exposed to a Storage Phosphor Screen (GE Healthcare), and scanned by a Storm 840 Molecular Imager (GE Healthcare). An autoradiogram is shown. Phosphotransfer between the HK domain of LOVHK and PhyR is shown on the left, and phosphotransfer between PAS-HK-NtrY and PhyR is shown on the right. Numbers above the autoradiogram indicate the time in seconds (columns 1 and 6) or in minutes (columns from 2 to 5 and columns 7 to 10).
(TIF)

S5 Fig Survival of *Brucella* strains in modified MM1 minimal medium. *B*. *abortus* 2308 wt (black) and the isogenic *lovhk*::*km* (blue), *∆lovR* (red) and *∆phyR* (green) mutant strains were grown in TSB medium up to logarithmic phase. First, an aliquot was drawn (time 0 h), then the rest of the culture was washed and resuspended in modified MM1 minimal medium, and aliquots were drawn at 1 h, 2 h, 10 h and 24 h. Viability was determined by plating aliquots of serial dilutions on TSA agar plates, and counting colony-forming units. The fraction of surviving cells for each strain after the indicated time of incubation in modified MM1 minimal medium was calculated (S/S_0_). Data represent mean survival ± standard deviation of two biological samples from one representative experiment. Data are presented in a semilog plot.
(TIF)

S6 Fig The GSR system in *Brucella abortus* wt is induced by starvation. *B*. *abortus* 2308 wt was grown in Minimal Medium—MM (modified Gerhardt-Wilson synthetic medium: 7.6 mM NH_4_SO_4_, 33 mM KH_2_PO_4_, 60.3 mM K_2_HPO_4_, 1.7 mM Na citrate, 1 mM MgSO_4_, 0.1% w/v yeast extract, 10 mM glucose, 2 μg ml^-1^ B6 vitamin, 2 μg ml^-1^ B1 vitamin and 1.22 μg ml^-1^ biotin, pH 7.0) [66], up to early logarithmic phase. First, an aliquot was drawn (time 0 h), then the rest of the culture was washed and resuspended in modified MM1 minimal medium, and an aliquot was drawn at 2 h. Expression of *phyR* gene was analyzed by qRT-PCR. The *if-1* housekeeping gene was used as a reference. The experiment was repeated twice with similar results. Data are reported as fold induction relative to wt at 0 h in modified MM1 minimal medium ± standard error of triplicate samples from one representative experiment. Statistical analysis between both treatments was assessed by a two-tail Student´s t-test (** = p<0.01).
(TIF)

S7 Fig Growth curve in rich medium. *B*. *abortus* 2308 wt (black) and the isogenic *lovhk*::*km* (blue), *∆lovR* (red), *∆phyR* (green) and *lovhk*::*km*/pMR_*lovhk* (orange) strains were transformed with pBBR-prom-*virB*-*lacZ* replicative vector, and the wt strain transformed with pBBR-*lacZ* empty vector (wt + e.v.) (grey). A culture of each strain was diluted to an initial OD_600_ of 0.05 in TSB and cultivated up to stationary phase. The OD_600_ was measured at the indicated time points. Data are presented in a semilog plot.
(TIF)

S1 Table List of strains and plasmids used in this study. (DOC)

S2 Table List of primers used in this study. (DOC)

S3 Table Proteins containing receiver domains encoded in the *B*. *abortus* 2308 genome and their interaction partners. (DOC)

S4 Table Pairwise percent identities of single domain RRs associated with the GSR response. (DOC)
