# Supplementary figures and images for: LOV Histidine Kinase Modulates the General Stress Response System and Affects the virB Operon Expression in Brucella abortus
Source: PLoS One. 2015 May 19;10(5):e0124058. doi: 10.1371/journal.pone.0124058 (PMC4438053; doi:10.1371/journal.pone.0124058)

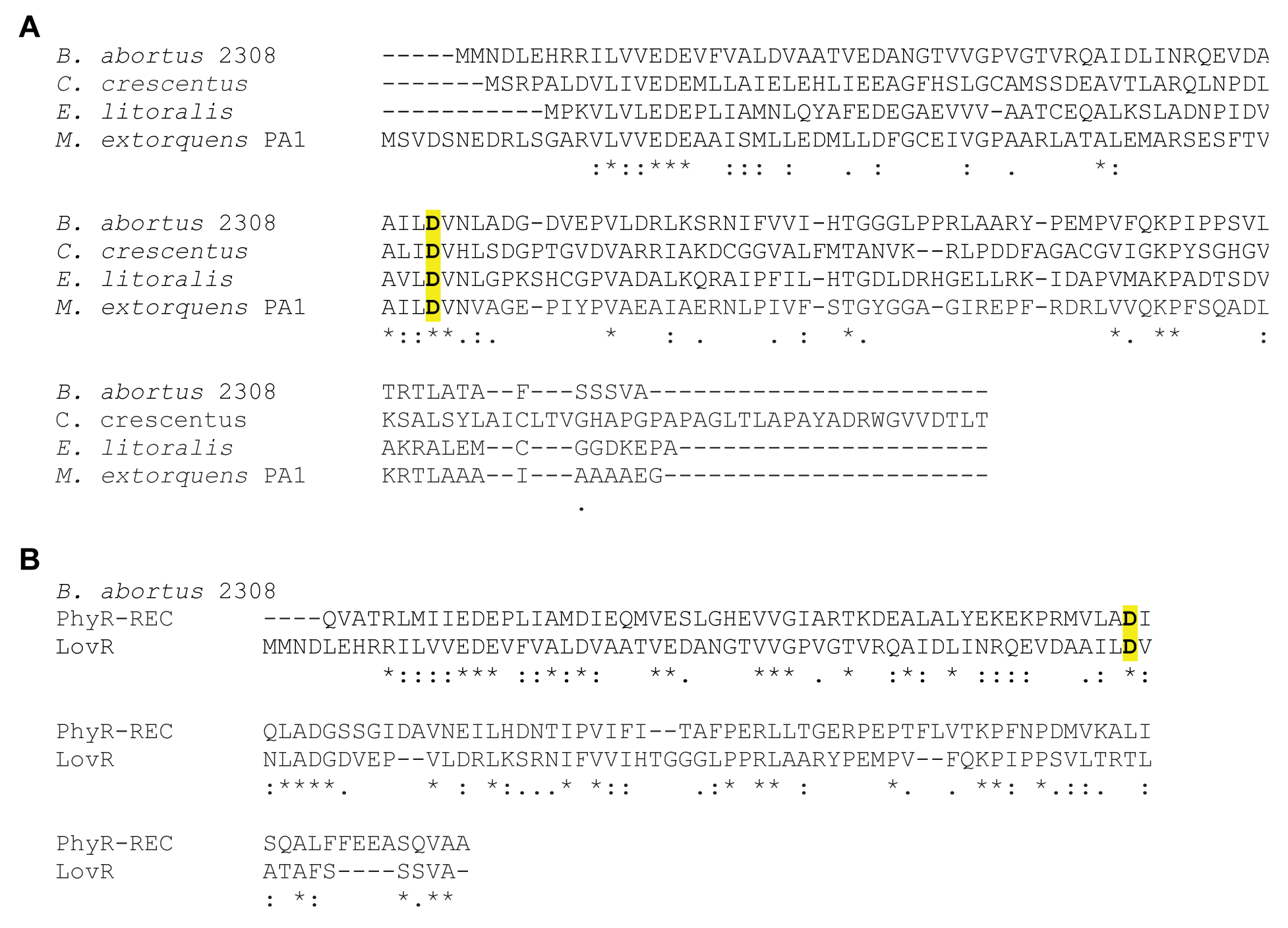

Supplement: S1 Fig — A. Alignment of LovR sequences of Brucella abortus 2308, Caulobacter crescentus CB15 [14], Erythrobacter litoralis HTCC2594 [15], and Mext_0407 from Methylobacterium extorquens PA1 [64] is shown. B. Alignment of Brucella abortus LovR and PhyR sequences is shown. In both cases, alignments were carried out with Clustal-Omega2 (EMBL-EBI) [65] and the predicted phosphorylatable aspartic acid is highlighted in yellow. References: ‘*’ fully conserved residue, ‘:’ conservation between groups of strongly similar properties, ‘.’ conservation between groups of weakly similar properties. (TIF) [file pone.0124058.s001.tif]

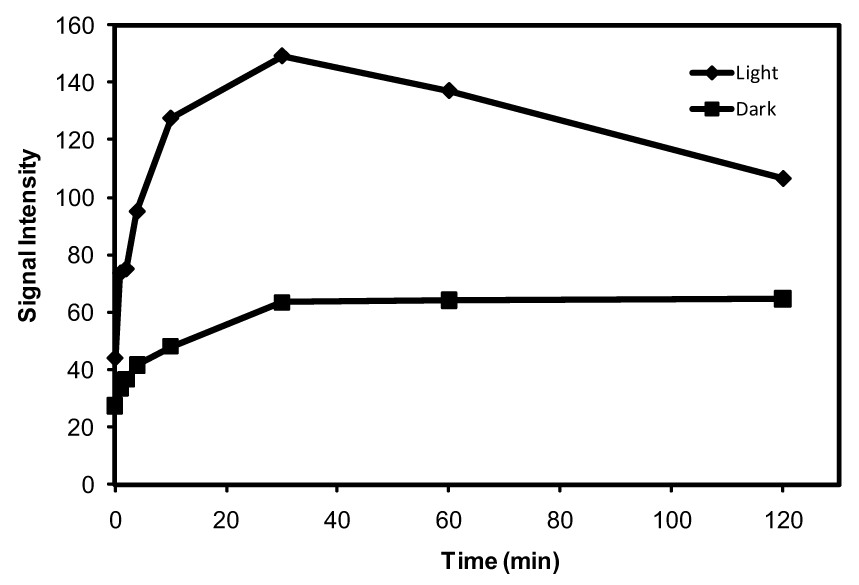

Supplement: S2 Fig — Autophosphorylation of LOVHK was carried out in 10 μl of final volume in phosphorylation buffer (20 mM Tris-HCl pH 8, 50 mM KCl and 5 mM MgCl2), containing 7.5 μl of purified LOVHK (5 μM) and 1 μCi of [γ-32P] ATP (111TBq/mmol, Perkin Elmer Life Sciences) that had been diluted 10x with cold 10 μM ATP. For the phosphorylation time course, samples were irradiated (light) for one minute of white light (a fluence of 2000 μmol m-2 s-1 white light; Kodak carousel 4400 projector) or mock-irradiated (dark), and incubation was continued at room temperature under dark conditions. Samples were taken at the indicated times and the reactions were stopped with an equal volume of 2X Laemmli sample buffer prior to SDS-PAGE (15%). The gel was dried and exposed to a phosphor screen (Typhoon) for quantitation. The experiment was done three times with results similar to those shown. (TIF) [file pone.0124058.s002.tif]

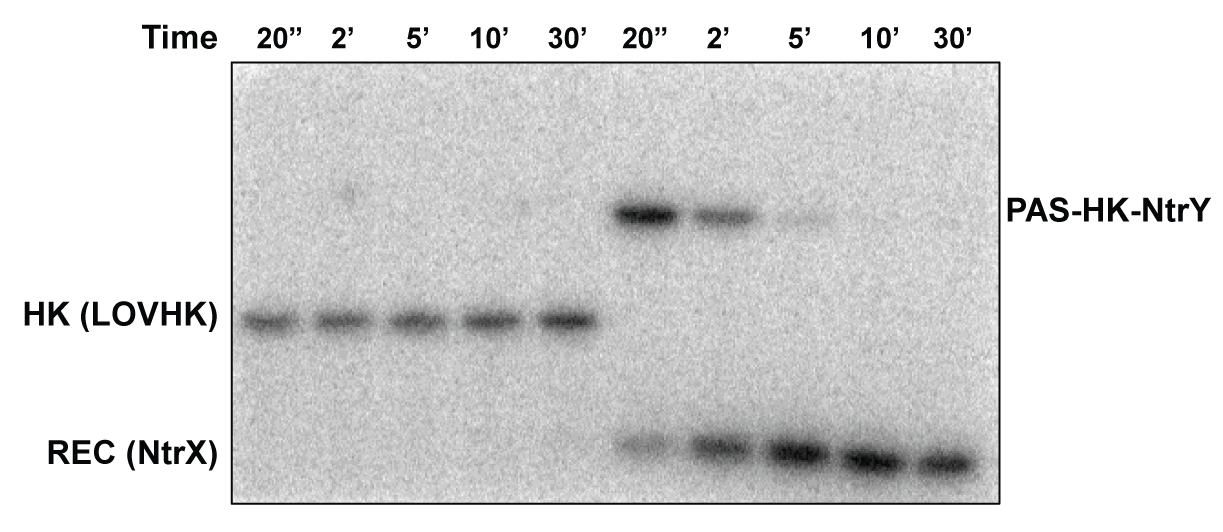

Supplement: S3 Fig — Purified recombinant HK domain of LOVHK (26 kDa) or PAS-HK-NtrY (42 kDa) at a concentration of 2.5 μM and 10 μM respectively, were incubated in phosphorylation buffer containing [γ-32P] ATP (separately). After 15 min at 37°C, the purified recombinant REC domain of NtrX (16 kDa) was added to each reaction mixture to a final ratio Histidine Kinase:NtrX 1:3. Aliquots were drawn at the indicated times and separated by 15% SDS-PAGE, exposed to a Storage Phosphor Screen (GE Healthcare), and scanned by a Storm 840 Molecular Imager (GE Healthcare). An autoradiogram is shown. Phosphotransfer between the HK domain of LOVHK and the REC domain of NtrX is shown on the left, and phosphotransfer between PAS-HK-NtrY and the REC domain of NtrX is shown on the right. Numbers above the autoradiogram indicate the time in seconds (columns 1 and 6) or in minutes (columns from 2 to 5 and columns 7 to 10). (TIF) [file pone.0124058.s003.tif]

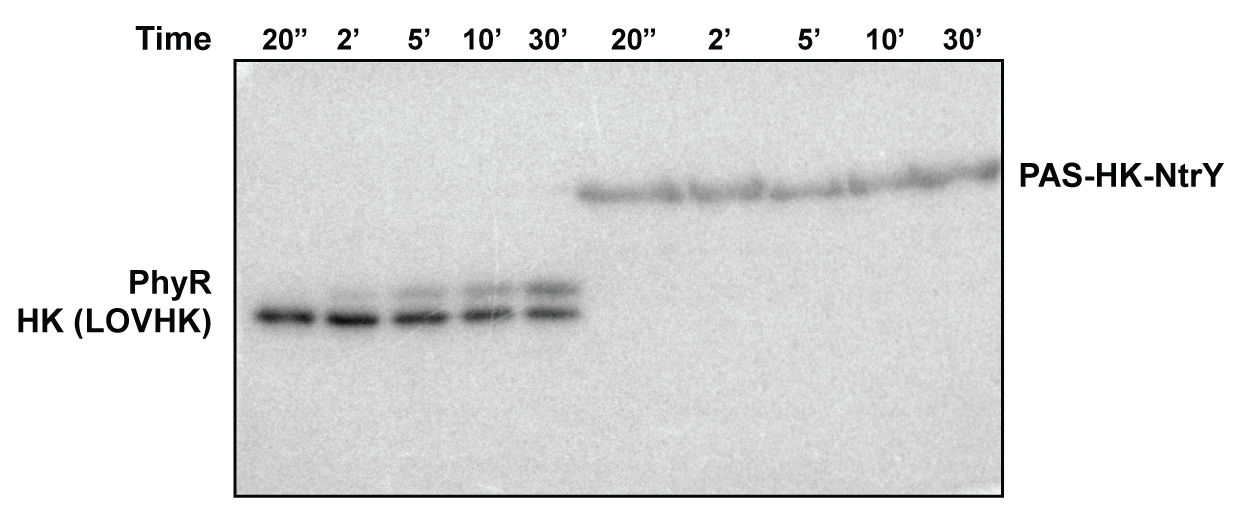

Supplement: S4 Fig — Purified recombinant HK domain of LOVHK (26 kDa) or PAS-HK-NtrY (42 kDa) at a concentration of 2.5 μM and 10 μM respectively, were incubated in phosphorylation buffer containing [γ-32P] ATP (separately). After 15 min at 37°C, purified recombinant PhyR (30 kDa) was added to each reaction mixture to a final concentration of 2.5 μM. Aliquots were drawn at the indicated times and separated by 15% SDS-PAGE, dried and exposed to a Storage Phosphor Screen (GE Healthcare), and scanned by a Storm 840 Molecular Imager (GE Healthcare). An autoradiogram is shown. Phosphotransfer between the HK domain of LOVHK and PhyR is shown on the left, and phosphotransfer between PAS-HK-NtrY and PhyR is shown on the right. Numbers above the autoradiogram indicate the time in seconds (columns 1 and 6) or in minutes (columns from 2 to 5 and columns 7 to 10). (TIF) [file pone.0124058.s004.tif]

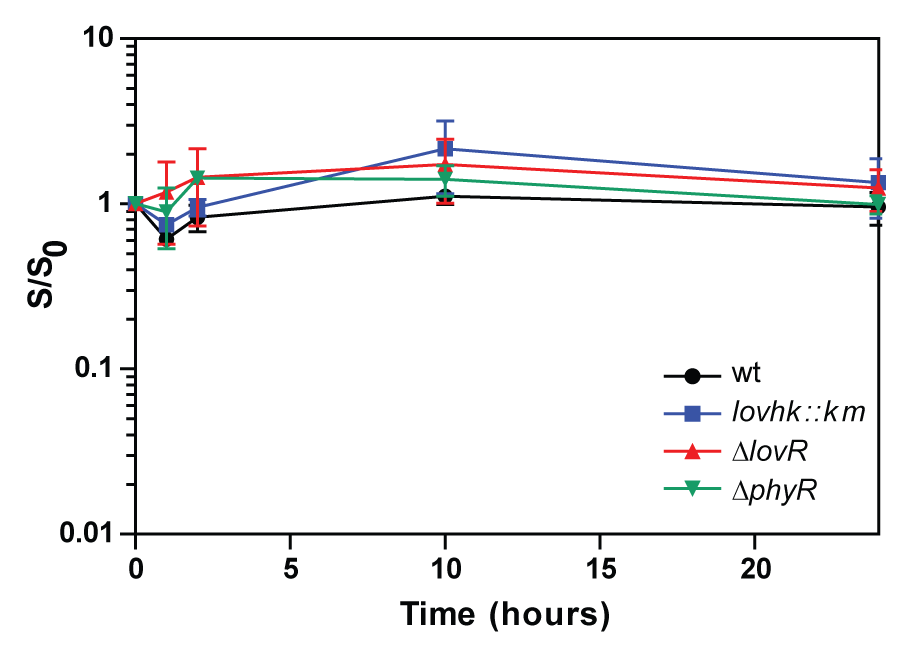

Supplement: S5 Fig — B. abortus 2308 wt (black) and the isogenic lovhk::km (blue), ∆lovR (red) and ∆phyR (green) mutant strains were grown in TSB medium up to logarithmic phase. First, an aliquot was drawn (time 0 h), then the rest of the culture was washed and resuspended in modified MM1 minimal medium, and aliquots were drawn at 1 h, 2 h, 10 h and 24 h. Viability was determined by plating aliquots of serial dilutions on TSA agar plates, and counting colony-forming units. The fraction of surviving cells for each strain after the indicated time of incubation in modified MM1 minimal medium was calculated (S/S0). Data represent mean survival ± standard deviation of two biological samples from one representative experiment. Data are presented in a semilog plot. (TIF) [file pone.0124058.s005.tif]

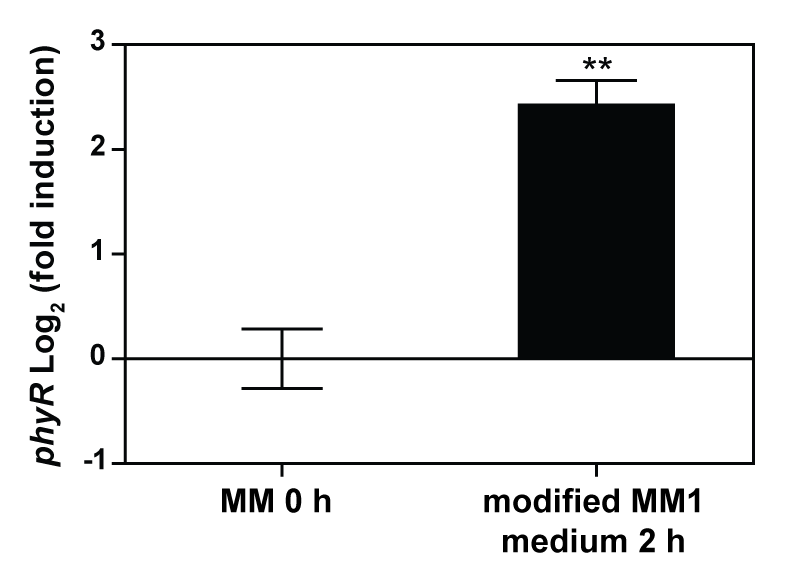

Supplement: S6 Fig — B. abortus 2308 wt was grown in Minimal Medium—MM (modified Gerhardt-Wilson synthetic medium: 7.6 mM NH4SO4, 33 mM KH2PO4, 60.3 mM K2HPO4, 1.7 mM Na citrate, 1 mM MgSO4, 0.1% w/v yeast extract, 10 mM glucose, 2 μg ml-1 B6 vitamin, 2 μg ml-1 B1 vitamin and 1.22 μg ml-1 biotin, pH 7.0) [66], up to early logarithmic phase. First, an aliquot was drawn (time 0 h), then the rest of the culture was washed and resuspended in modified MM1 minimal medium, and an aliquot was drawn at 2 h. Expression of phyR gene was analyzed by qRT-PCR. The if-1 housekeeping gene was used as a reference. The experiment was repeated twice with similar results. Data are reported as fold induction relative to wt at 0 h in modified MM1 minimal medium ± standard error of triplicate samples from one representative experiment. Statistical analysis between both treatments was assessed by a two-tail Student´s t-test (** = p<0.01). (TIF) [file pone.0124058.s006.tif]

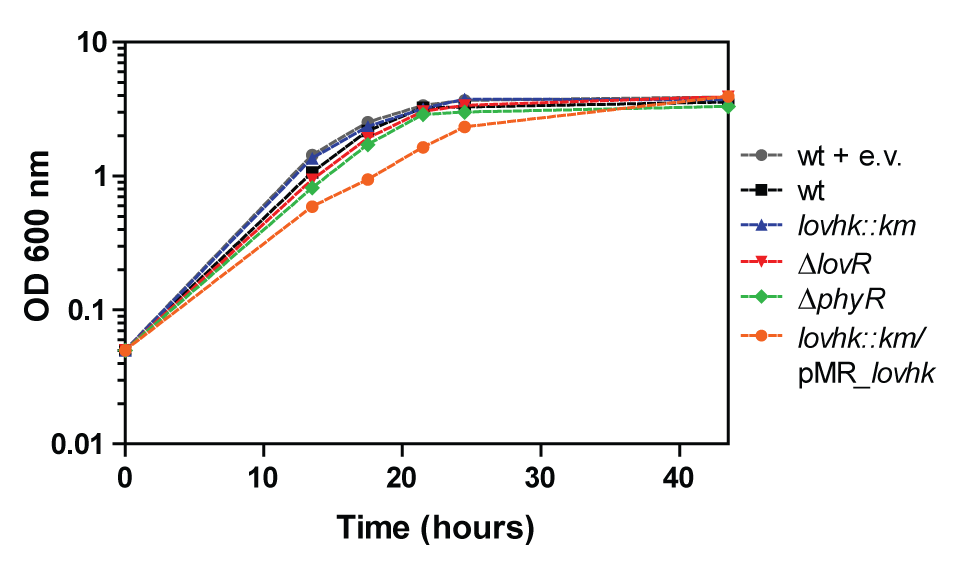

Supplement: S7 Fig — B. abortus 2308 wt (black) and the isogenic lovhk::km (blue), ∆lovR (red), ∆phyR (green) and lovhk::km/pMR_lovhk (orange) strains were transformed with pBBR-prom-virB-lacZ replicative vector, and the wt strain transformed with pBBR-lacZ empty vector (wt + e.v.) (grey). A culture of each strain was diluted to an initial OD600 of 0.05 in TSB and cultivated up to stationary phase. The OD600 was measured at the indicated time points. Data are presented in a semilog plot. (TIF) [file pone.0124058.s007.tif]
