# Supplementary material for: LOV Histidine Kinase Modulates the General Stress Response System and Affects the virB Operon Expression in Brucella abortus
Source: PLoS One. 2015 May 19;10(5):e0124058. doi: 10.1371/journal.pone.0124058 (PMC4438053; doi:10.1371/journal.pone.0124058)
Supplement: S1 Table — (DOC) [file pone.0124058.s008.doc]

**S1 Table.** **List of strains and plasmids used in this study.**

| **Strain or plasmid** | **Description and/or relevant phenotype** | **Reference** |
| --- | --- | --- |
| ***Strains*** |  |  |
| ***E. coli*** |  |  |
| DH5α | *Lac*ZΔM15 *recA1 hsdR17 supE44 thi-1 gyrA relA1* | Invitrogen |
| S17-1 | Movilization host: cells enable movilizaton of pBBR plasmids from *E. coli* S17-1 to *Brucella* strains. *recA, thi, pro, hsdR^-^M^-^* RP4 : 2-Tc:Mu: Km Tn7 λpir. | *Escherichia coli* (ATCC® 47055™) |
| BL21(DE3)pLysS | cells enable high-level expression of heterologous proteins in *E. coli* | Stratagene |
| DH5α F’IQ Km^R^ | used for amplification of pBT and pTRG plasmids and library construction | Life Technologies |
| BacterioMatch® II Two-Hybrid System Reporter Cells | F*´ lacIq HIS3 aadA* Kan*r*. This strain contains the *HIS*3-*aadA* reporter cassette. Detection of protein-protein interactions is based on transcriptional activation of the *HIS3* reporter gene, which allows growth in the presence of 3-amino-1,2,4- triazole (3-AT), a competitive inhibitor of His3 enzyme. Positives are verified by using the *aadA* gene, which confers streptomycin resistance, as a secondary reporter. | Stratagene |
| FW 102 O_L_2-62 | fusion where the λCI operator is centered at position -62 upstream of the lac promoter |  |
| ***Brucella abortus*** |  |  |
| wt 2308 | Wild-type, smooth, virulent, Nal^R^ | Laboratory stock |
| *lovhk::km* | 2308, Km^R^, insertion of km cassette in *lovhk* (BAB2_0652) |  |
| *lovhk::km/*pMR_*lovhk* | *lovhk::km* mutant strain complemented with the pMR10*cat*_*lovhk* vector*,* which expresses *lovhk* under the control of its own promoter | This work |
| *ΔlovR* | 2308, deletion of *lovR* (BAB1_0099) | This work |
| *ΔphyR* | 2308, deletion of *phyR* (BAB1_1671) | This work |
| ***Plasmids*** |  |  |
| pET24A | Km^R^. Plasmid for the overexpression of His6-tagged proteins in *E. coli* | Novagen |
| pET24A-LOVHK | Km^R^. Plasmid for the overexpression of His6-tagged full length LOVHK | This work |
| pET24A-HK | Km^R^. Plasmid for the overexpression of His6-tagged HK domain of LOVHK | This work |
| pTrcHisB | Amp^R^. Plasmid for the overexpression of His6-tagged proteins in *E. coli* | Invitrogen |
| pTrcHisB-LovR | Amp^R^. Plasmid for the overexpression of His6-tagged LovR | This work |
| pGEM-T Easy | Amp^R^. Plasmid for cloning | Promega |
| pGEM-PhyR | Amp^R^. Derivative of pGEM-T Easy containing the sequence of His6-tagged PhyR | This work |
| pET24A-PhyR | Km^R^. Plasmid for the overexpression of His6-tagged PhyR | This work |
| pET24D | Km^R^. Plasmid for the overexpression of His6-tagged proteins in *E. coli* | Novagen |
| pET24D-PAS-HK-NtrY | Km^R^. Plasmid for the overexpression of His6-tagged PAS plus histidine kinase fragment of NtrY |  |
| pBT | Cm^R^. 52 bp *MCS*, 3.2 kb size, *MCS, p15A* origin of replication, *lac-UV5*, λ c1 ORF | Stratagene |
| pTRG | Tet^R^. 60 bp *MCS*, 4.4 kb size, *MCS, lac-UV5* promoter, *ColE1* origin of replication, *RNAPα ORF* | Stratagene |
| pBT-LGF2 | Interaction control plasmid encoding the dimerization domain of the Gal4 transcriptional activator protein in fusion with λCI | Stratagene |
| pTRG-Gal11 | Interaction control plasmid encoding a domain of the mutant form of the Gal11 protein in fusion with α-RNAP | Stratagene |
| pBT-LOVHK | Derivative of pBT in which full length LOVHK was cloned in-frame with λCI | This work |
| pBT-PdhS | Derivative of pBT in which PdhS was cloned in-frame with λCI | This work |
| pTRG-DivK | Derivative of pTRG in which DivK was cloned in-frame to α-RNAP | This work |
| pTRG-LovR | Derivative of pTRG in which LovR was cloned in-frame to α-RNAP | This work |
| pTRG-MdrR | Derivative of pTRG in which MdrR was cloned in-frame to α-RNAP | This work |
| pTRG-OtpR | Derivative of pTRG in which the REC domain of OtpR was cloned in-frame to α-RNAP | This work |
| pTRG-PhoB | Derivative of pTRG in which the REC domain of PhoB was cloned in-frame to α-RNAP | This work |
| pTRG-FeuP | Derivative of pTRG in which the REC domain of FeuP was cloned in-frame to α-RNAP | This work |
| pTRG-CtrA | Derivative of pTRG in which the REC domain of CtrA was cloned in-frame to α-RNAP | This work |
| pTRG-TccR | Derivative of pTRG in which the REC domain of TccR was cloned in-frame to α-RNAP | This work |
| pTRG-BvrR | Derivative of pTRG in which the REC domain of BvrR was cloned in-frame to α-RNAP | This work |
| pTRG-TcbR | Derivative of pTRG in which the REC domain of TcbR was cloned in-frame to α-RNAP | This work |
| pTRG-TcfR | Derivative of pTRG in which the REC domain of TcfR was cloned in-frame to α-RNAP | This work |
| pTRG-TceR | Derivative of pTRG in which the REC domain of TceR was cloned in-frame to α-RNAP | This work |
| pTRG-FtcR | Derivative of pTRG in which the REC domain of FtcR was cloned in-frame to α-RNAP | This work |
| pTRG-PrlR | Derivative of pTRG in which the REC domain of PrlR was cloned in-frame to α-RNAP | This work |
| pTRG-TcdR | Derivative of pTRG in which the REC domain of TcdR was cloned in-frame to α-RNAP | This work |
| pTRG-NodW | Derivative of pTRG in which the REC domain of NodW was cloned in-frame to α-RNAP | This work |
| pTRG-NtrX | Derivative of pTRG in which the REC domain of NtrX was cloned in-frame to α-RNAP |  |
| pTRG-NtrC | Derivative of pTRG in which the REC domain of NtrC was cloned in-frame to α-RNAP | This work |
| pTRG-StcA | Derivative of pTRG in which the REC domain of StcA was cloned in-frame to α-RNAP | This work |
| pTRG-PrrA | Derivative of pTRG in which the REC domain of PrrA was cloned in-frame to α-RNAP |  |
| pTRG-PleD | Derivative of pTRG in which the REC domain of PleD was cloned in-frame to α-RNAP | This work |
| pTRG-PhyR | Derivative of pTRG in which the REC domain of PhyR was cloned in-frame to α-RNAP | This work |
| pTRG-BAB1_0806 | Derivative of pTRG in which the REC domain of BAB1_0806 was cloned in-frame to α-RNAP | This work |
| pk18mobsacB | Km^R^. Mobilizable an suicide vector in *Brucella*, containing the counterselection marker sacB |  |
| pk18mobsacB*_∆lovR* | Derivative of pk18mobsacB containing the flanking regions of *lovR* for *∆lovR* mutant strain construction | This work |
| pk18mobsacB*_∆phyR* | Derivative of pk18mobsacB containing the flanking regions of *phyR* for *∆phyR* mutant strain construction | This work |
| pMR10*cat* | Cm^R^ and Km^R^. RK2*ori*V-based low-copy-number plasmid derived from pMR10. Broad-host-range plasmid, replicative in *Brucella* | A kind gift of Dr. Xavier De Bolle (C. D. Mohr and R. C. Roberts: Unpublished data) |
| pMR10*cat*_*lovhk* | Derivative of pMR10*cat* containing the *lovhk* gene and a 581 bp fragment upstream of *lovhk* | This work |
| pBBR1MSC-4 | Amp^R^. pBBR1MCS-4 Broad-host-range cloning vector |  |
| pBBR-*lacZ* | pBBR1MCS-4 with a 3.5 kb fragment that contains a *lacZ* promoter-probe cassette from plasmid pAB1002 | This work |
| pBBR-prom-*virB*-lacZ | pBBR-*lacZ* with a 456 pb fragment upstream of *B. abortus* *virB* operon | This work and |

**References**

1. Deaconescu AM, Chambers AL, Smith AJ, Nickels BE, Hochschild A, et al. (2006) Structural basis for bacterial transcription-coupled DNA repair. Cell 124: 507-520.

2. Swartz TE, Tseng TS, Frederickson MA, Paris G, Comerci DJ, et al. (2007) Blue-light-activated histidine kinases: two-component sensors in bacteria. Science 317: 1090-1093.

3. Carrica Mdel C, Fernandez I, Marti MA, Paris G, Goldbaum FA (2012) The NtrY/X two-component system of *Brucella* spp. acts as a redox sensor and regulates the expression of nitrogen respiration enzymes. Mol Microbiol 85: 39-50.

4. Carrica Mdel C, Fernandez I, Sieira R, Paris G, Goldbaum FA (2013) The two-component systems PrrBA and NtrYX co-ordinately regulate the adaptation of *Brucella abortus* to an oxygen-limited environment. Mol Microbiol 88: 222-233.

5. Schafer A, Tauch A, Jager W, Kalinowski J, Thierbach G, et al. (1994) Small mobilizable multi-purpose cloning vectors derived from the *Escherichia coli* plasmids pK18 and pK19: selection of defined deletions in the chromosome of *Corynebacterium glutamicum*. Gene 145: 69-73.

6. Kovach ME, Phillips RW, Elzer PH, Roop RM, 2nd, Peterson KM (1994) pBBR1MCS: a broad-host-range cloning vector. Biotechniques 16: 800-802.

7. Sieira R, Comerci DJ, Pietrasanta LI, Ugalde RA (2004) Integration host factor is involved in transcriptional regulation of the *Brucella abortus virB* operon. Mol Microbiol 54: 808-822.
