# Supplementary material for: LOV Histidine Kinase Modulates the General Stress Response System and Affects the virB Operon Expression in Brucella abortus
Source: PLoS One. 2015 May 19;10(5):e0124058. doi: 10.1371/journal.pone.0124058 (PMC4438053; doi:10.1371/journal.pone.0124058)
Supplement: S2 Table — (DOC) [file pone.0124058.s009.doc]

**S2 Table**. List of primers used in this study.

| **Oligonucleotides name** | **5’ to 3’ sequences** | **Restriction Site** |
| --- | --- | --- |
| ***Cloning of LOVHK and PdhS genes in pBT for the two hybrid assays*** | |  |
| Full_LOV_forward | AGAGCGGCCGCAATGGCAATAGATTTAAGGCCATTC | NotI |
| Full_LOV_reverse | AGACTCGAGTCACGCGATGCGCTGTTCCG | XhoI |
| PdhS_c-ter_forward | AGAGCGGCCGCAGAGGAAAAGCAGGCGCTCG | NotI |
| PdhS_c-ter_reverse | AGACTCGAGTCAGTCAGCCAGAACGCGG | XhoI |
| ***Cloning of response regulator REC domains in pTRG for the two hybrid assays*** | |  |
| BAB1_0099_forward | AGAGCGGCCGCATTGATGAACGATCTCGAACATAGG | NotI |
| BAB1_0099_reverse | AGACTCGAGTCAGGCGACGCTGGATGAAAA | XhoI |
| BAB1_0136_forward | AGAGCGGCCGCAATGGAAGACTTGAAGCAGGAAGA | NotI |
| BAB1_0136_reverse | AGACTCGAGCGCTTGGCGAGGATGCGTTG | XhoI |
| BAB1_0345_forward | AGAGCGGCCGCAATGCATTTCATTATCGCTGATGA | NotI |
| BAB1_0345_reverse | AGACTCGAGTCAAACGCCCAGAACGTCGC | XhoI |
| BAB1_0370_forward | AGAGCGGCCGCAATGACGAATGAATGCAAGATTCG | NotI |
| BAB1_0370_reverse | AGACTCGAGCTAAAGTGAAGACAGGCACAGCC | XhoI |
| BAB1_0628_forward | AGAGCGGCCGCATTGCGTATCCTGATTGTTGAAG | NotI |
| BAB1_0628_reverse | AGACTCGAGTCAGCTGCCGCTCCCTTTTGC | XhoI |
| BAB1_0636_forward | AGAGCGGCCGCAATGAAAATTCTCGTTATCGAAGATGA | NotI |
| BAB1_0636_reverse | AGACTCGAGTCATTCCGCCCTTGCGGCAG | XhoI |
| BAB1_1138_forward | AGAGCGGCCGCAATGGCGGCCGATATTCTTGTT | NotI |
| BAB1_1138_reverse | AGACTCGAGTTATACGCCGAGAGACTTCAGC | XhoI |
| BAB1_1140_forward | AGAGCGGCCGCAATGATTGCAGGGCGTCCGAC | NotI |
| BAB1_1140_reverse | AGACTCGAGCTAACCGCCGGTCTTTGCGC | XhoI |
| BAB1_1538_forward | AGAGCGGCCGCAATGGGATCGAAAGACGCCAT | NotI |
| BAB1_1538_reverse | AGACTCGAGCTATTCGATGCTGAGTTTGTAGCC | XhoI |
| BAB1_1614_forward | AGAGCGGCCGCAATGCGCGTCCTTTTGATTG | NotI |
| BAB1_1614_reverse | AGACTCGAGTCAGGCGCTTTCGCGCATCT | XhoI |
| BAB1_1671_forward | AGAGCGGCCGCAATGACGTTATCGACGCGTATAGC | NotI |
| BAB1_1671_reverse | AGACTCGAGTCAGGCAGCAACTTGCGATG | XhoI |
| BAB1_2006_forward | AGAGCGGCCGCAATGACAGGCCGTACTATTCTGATT | NotI |
| BAB1_2006_reverse | AGACTCGAGTTATGGGACAAGCTTATATCCCC | XhoI |
| BAB1_2092_forward | AGAGCGGCCGCAATGAAGGAAGCTTCGGCAA | NotI |
| BAB1_2092_reverse | AGACTCGAGTTACGCTTCCCGGAAACGAT | XhoI |
| BAB1_2146_forward | AGAGCGGCCGCAATGTCCCAAGTACCCCGAA | NotI |
| BAB1_2146_reverse | AGACTCGAGTCAGCCGAACGAATATCCC | XhoI |
| BAB2_0041_forward | AGAGCGGCCGCAATGAACTGCGCATATGAACGC | NotI |
| BAB2_0041_reverse | AGACTCGAGTCAAAGCTCTTTGACCGGTTG | XhoI |
| BAB2_0042_forward | AGAGCGGCCGCAATGAAGAGAATCCTTCTAGCTGAAGA | NotI |
| BAB2_0042_reverse | AGACTCGAGTCAGGCGGCGATCAGCATTTTTTC | XhoI |
| BAB2_0081_forward | AGAGCGGCCGCAATGGCAACGCGCATTCTCAT | NotI |
| BAB2_0081_reverse | AGACTCGAGCTATATGGCCGCGCGGCCCCA | XhoI |
| BAB2_0222_forward | AGAGCGGCCGCAATGAGAATTATCCTCATCGAAGACG | NotI |
| BAB2_0222_reverse | AGACTCGAGTCATCGTGCTGCTTTCGATTTG | XhoI |
| BAB2_0628_forward | AGAGCGGCCGCAATGACGAAAAGCGTAATGATCG | NotI |
| BAB2_0628_reverse | AGACTCGAGCTAGGCATCGCCCAGATAGG | XhoI |
| BAB2_0630_forward | AGAGGATTCATGACAGCAAGGATTCTCGTCG | EcoRI |
| BAB2_0630_reverse | AGACTCGAGTCAGGCCGCGGATAAAACAAT | XhoI |
| BAB2_0762_forward | AGAGCGGCCGCATTGAAAGAAGACGCCCACA | NotI |
| BAB2_0762_reverse | AGACTCGAGTCATGCTATTTCCACTTCATGC | XhoI |
| BAB2_0806_forward | AGAGCGGCCGCAATGAATATTTCTCCACAGTTCAAAGATG | NotI |
| BAB2_0806_reverse | AGACTCGAGCTATTGCAGGTCGGAAAACG | XhoI |
| BAB2_1099_forward | AGAGCGGCCGCAATGATTGTTGTCGTTGACGACA | NotI |
| BAB2_1099_reverse | AGACTCGAGTCACTCGATATTGATGCAGTAGCC | XhoI |
| ***Cloning of Brucella genes for expression of recombinant proteins*** | |  |
| LOVHK_Full_NheI_Fw | GCGATGGCTAGCGAATTCACGCTTATGCCCATG | NheI |
| LOVHK_Full_XhoI_Rev | GCGCTCGAGACTACCGCGTGGCACCAGAGCCGCGATGCGCTGTTCCGGCAC | XhoI |
| BaLOV_5Dhpt_NheI | TTAGGATCCGCTAGCgtgcaggatgtaaccgag | NheI |
| BaLOV_3CAL_SalI | TTAGGATCCGTCGACcgtgattggggccagaacgt | SalI |
| LovR_FWD_NheI | CGCGCTAGCTTGATGAACGATCTCGAACATAGG | NheI |
| LovR_REV_XhoI | agactcgagtcaggcgacgctggatgaaaa | XhoI |
| 1671_NdeI_FWD | atacatATGACGTTATCGACGCGTATA | NdeI |
| 1671_His_Stop_REV | TTAGGATCCtcaatgatgatgatgatgatgGGCAGCAACTTGCGATG | ---- |
| ***Brucella abortus 2308 mutant and complemented strains construction*** | |  |
| 5LovR_FWD-BamHI | CGCGGATCCACTGGCGCTTTGTCGTGAA | BamHI |
| 5LovR_REV | GGATGAAGATCGTTCATCAATTTTTTCTGCT | ---- |
| 3LovR_FWD | GAACGATCTTCATCCAGCGTCGCCTGATG | ---- |
| 3LovR_REV-SalI | CGCGTCGACGTTCGCACCGCGCCCGAT | SalI |
| ΔBAB1_1671(1)_EcoRI_Fw | TTAgaattcCTCCTGTTCGGCTTGGTCGAG | EcoRI |
| ΔBAB1_1671(2)_BamHI_Rev | gaacgaggatcctccCGATAACGTCATGATTC | BamHI |
| ΔBAB1_1671(3)_BamHI_Fw | ggaggatcctcgttcGTTGCTGCCTGACG | BamHI |
| ΔBAB1_1671(4)_PstI_Fw | tatctgcagGTGCCCGCTTGTCCCTGACTG | PstI |
| pMR10_pLOVHK_F | GCCAAGCTTCCATGGGATATCGAGAACCGCACTAACTATTTGTTTTGTC | ---- |
| pMR10_pLOVHK_R | CGTAACTTAAGTGCGGCCCCCTCGAGATAGCCATGGCGGTAATAACGAT | ---- |
| ***Real Time quantitative RT-PCR assays*** | |  |
| qPCR_0282_IF1_Fw (2) | tgttacggaactgctgcccaat | ---- |
| qPCR_0282_IF1_Rev (2) | cggcccttggtcaggtcataa | ---- |
| LOV_bmf_RT_F | cgacgtgacattggagctta | ---- |
| LOV_bmf_RT_R | aatcaagctggtccttgctg | ---- |
| LovR_bmf_RT_F | gccgaaatatcttcgtggtc | ---- |
| LovR_bmf_RT_R | gtatcggcttctggaaaacc | ---- |
| qPCR_1671_Fw (2) | caggtggcaacacgtctgatgat | ---- |
| qPCR_1671_Rev (2) | gctggatatcggcgagaaccat | ---- |
| rpoH1_RT_FW | AGTTCAAGCTGCCGATGAGT | ---- |
| rpoH1_RT_REV | CCAGGATGCATAGGTCGAAA | ---- |
| dps_RT_FW | ATCTTGCCCTCATCACCAAG | ---- |
| dps_RT_REV | CACATGGTCGTCGAGTTCTG | ---- |
| ***pBBR_prom_LacZ construction*** |  |  |
| pvirbup | GCGGATCCATGACAGGCATATTTCAAC | BamHI |
| pvirbdown | ATGAATTCGATCGTCTCTTCTCAGA | EcoRI |

**References**

1. Kim HS, Caswell CC, Foreman R, Roop RM, 2nd, Crosson S. The *Brucella abortus* general stress response system regulates chronic mammalian infection and is controlled by phosphorylation and proteolysis. The Journal of biological chemistry. 2013;288(19):13906-16. Epub 2013/04/03. doi: 10.1074/jbc.M113.459305. PubMed PMID: 23546883; PubMed Central PMCID: PMC3650426.

2. Sieira R, Comerci DJ, Pietrasanta LI, Ugalde RA. Integration host factor is involved in transcriptional regulation of the *Brucella abortus* virB operon. Molecular microbiology. 2004;54(3):808-22. Epub 2004/10/20. doi: 10.1111/j.1365-2958.2004.04316.x. PubMed PMID: 15491369.
