# Supplementary material for: LOV Histidine Kinase Modulates the General Stress Response System and Affects the virB Operon Expression in Brucella abortus
Source: PLoS One. 2015 May 19;10(5):e0124058. doi: 10.1371/journal.pone.0124058 (PMC4438053; doi:10.1371/journal.pone.0124058)
Supplement: S3 Table — (DOC) [file pone.0124058.s010.doc]

**S3 Table. Proteins containing receiver domains encoded in the *B. abortus*** 2308 genome and their interaction partners.

| **Response^1^**  **Regulator** | **Name^2^** | **Interaction^2^** | **Operon** | **Function** |
| --- | --- | --- | --- | --- |
| **REC only** |  |  |  |  |
| BAB1_0099 | LovR | yes^3^ (LOVHK) | no | Phosphate sink for LOVHK^3^. |
| BAB2_0628 | DivK | yes (PdhS, PleC, DivL, DivJ) | no | Asymmetric division |
| BAB2_0042 | MdrR | unknown | no | unknown |
| **OmpR-type** |  |  |  |  |
| BAB1_2006 | OtpR/CenR | yes (TcbR) | no | Virulence, stress tolerance (temperature, acid media, hyperosmotic media), cell morphology, tolerance to β-lactam agents, and necessary for growth |
| BAB1_2146 | PhoB | yes (PhoR) | no | unknown |
| BAB1_0628 | FeuP | no | *feuPQ* | Unknown (It does not seem to be involved in regulation of iron uptake) |
| BAB1_1614 | CtrA | unknown | no | Transcriptional regulator, involved in cell division. Essential for cell viablility |
| BAB1_0636 | TccR | yes (TccS) | *tccSR* | unknown |
| BAB1_2092 | BvrR | yes (BvrS) | *bvrRS* | polycation resistance, virulence, cell invasion, vacuole maturation, intracellular trafficking and replication, modulates *virB* expression and Omp (outer membrane protein) expression |
| BAB1_1538 | TcbR | yes (OtpR/CenR) | *tcbSR* | Unknown |
| BAB2_0222 | TcfR | yes (TcfS) | *tcfSR* | Unknown |
| BAB2_0762 | TceR | yes (TceS, TceR) | *tceSR* | Unknown |
| BAB2_1099 | FtcR | no | no | Master regulator of the flagellar system, virulence. Absence of a classical phosphorylation site |
| **NarL-type** |  |  |  |  |
| BAB1_0345 | PrlR | PrlS | no (both genes are in the same genomic region, but are codified in opposite directions). | Persistence in mice and resistance to ionic strength |
| BAB1_0370 | TcdR | yes (TcdS) | *tcdSR* | Unknown |
| BAB2_0041 | NodW | yes (NodV) | *nodVW* | Unknown |
| **LuxR-type** |  |  |  |  |
| BAB2_0806 | LuxR-like | no | no | Unknown |
| **NtrC-type** |  |  |  |  |
| BAB1_1138 | NtrX | yes (NtrY) | *nrtYX* | Redox sensor. Regulation of denitrification pathway genes and high-affinity cytochrome oxidases. Important for macrophage infection . |
| BAB1_1140 | NtrC | no | *ntrBC* | Probably involved in regulation of nitrogen metabolism |
| BAB2_0081 | StcA | no | no | Unknown |
| **PrrA-type** |  |  |  |  |
| BAB1_0136 | PrrA/RegA | yes (PrrB/RegB) | no | Redox sensing. Regulation of denitrification pathway genes, high-affinity cytochrome oxidase genes, and genes involved in adaptation to low oxygen tension in *B. abortus* . Mouse infection persistence within oxygen-limited target organs, regulation the expression of genes involved in oxidative respiration and denitrification in *B. suis* . |
| **REC-GGDEF** |  |  |  |  |
| BAB2_0630 | PleD | no | no | Involved in the cell cycle regulation in *C. crescentus* |
| **RpoE-REC** |  |  |  |  |
| BAB1_1671 | PhyR | yes (LOVHK)^3^ | yes (BAB1_1669) | General Stress Response system, required for chronic mammalian infection,regulated by phosphorylation and proteolysis. |
| **Hybrid HisK** |  |  |  |  |
| ψBAB1_1059^4^ | Pseudogene  CckA | no | no | This gene is present in other *Brucella* species including *B. melitensis*, *B. abortus* 9-941, *B. suis* 1330 and *B. abortus* S-19 and encodes for CckA protein involved in the cell cycle regulation of *C. crescentus.* |
| BAB1_0346 | PrlS | PrlR | no | Persistence in mice and resistance to ionic strength |

^1^ Classification of response regulator families according to Galperin, 2006 .

^2^ Names of two component systems and interaction data were taken from Hallez *et al,* 2007 .

^3^ This work.

4 This gene was not included in the analysis since it does not encode for a protein in *B. abortus* 2308.

**References**

1. Hallez R, Mignolet J, Van Mullem V, Wery M, Vandenhaute J, Letesson JJ, et al. The asymmetric distribution of the essential histidine kinase PdhS indicates a differentiation event in *Brucella abortus*. The EMBO journal. 2007;26(5):1444-55. Epub 2007/02/17. doi: 10.1038/sj.emboj.7601577. PubMed PMID: 17304218; PubMed Central PMCID: PMC1817626.

2. Liu W, Dong H, Gao X, Zhang C, Wu Q. OtpR regulated the growth, cell morphology of *B. melitensis* and tolerance to beta-lactam agents. Veterinary microbiology. 2012;159(1-2):90-8. Epub 2012/04/17. doi: 10.1016/j.vetmic.2012.03.022. PubMed PMID: 22503392.

3. Zhang X, Ren J, Li N, Liu W, Wu Q. Disruption of the BMEI0066 gene attenuates the virulence of *Brucella melitensis* and decreases its stress tolerance. International journal of biological sciences. 2009;5(6):570-7. Epub 2009/09/11. PubMed PMID: 19742243; PubMed Central PMCID: PMC2737717.

4. Dorrell N, Spencer S, Foulonge V, Guigue-Talet P, O'Callaghan D, Wren BW. Identification, cloning and initial characterisation of FeuPQ in *Brucella suis*: a new sub-family of two-component regulatory systems. FEMS microbiology letters. 1998;162(1):143-50. Epub 1998/05/22. PubMed PMID: 9595675.

5. Bellefontaine AF, Pierreux CE, Mertens P, Vandenhaute J, Letesson JJ, De Bolle X. Plasticity of a transcriptional regulation network among alpha-proteobacteria is supported by the identification of CtrA targets in *Brucella abortus*. Molecular microbiology. 2002;43(4):945-60. Epub 2002/04/04. PubMed PMID: 11929544.

6. Sola-Landa A, Pizarro-Cerda J, Grillo MJ, Moreno E, Moriyon I, Blasco JM, et al. A two-component regulatory system playing a critical role in plant pathogens and endosymbionts is present in *Brucella abortus* and controls cell invasion and virulence. Molecular microbiology. 1998;29(1):125-38. Epub 1998/08/14. PubMed PMID: 9701808.

7. Martinez-Nunez C, Altamirano-Silva P, Alvarado-Guillen F, Moreno E, Guzman-Verri C, Chaves-Olarte E. The two-component system BvrR/BvrS regulates the expression of the type IV secretion system VirB in *Brucella abortus*. Journal of bacteriology. 2010;192(21):5603-8. Epub 2010/09/14. doi: 10.1128/JB.00567-10. PubMed PMID: 20833814; PubMed Central PMCID: PMC2953682.

8. Lopez-Goni I, Guzman-Verri C, Manterola L, Sola-Landa A, Moriyon I, Moreno E. Regulation of *Brucella* virulence by the two-component system BvrR/BvrS. Veterinary microbiology. 2002;90(1-4):329-39. Epub 2002/11/05. PubMed PMID: 12414153.

9. Guzman-Verri C, Manterola L, Sola-Landa A, Parra A, Cloeckaert A, Garin J, et al. The two-component system BvrR/BvrS essential for *Brucella abortus* virulence regulates the expression of outer membrane proteins with counterparts in members of the Rhizobiaceae. Proceedings of the National Academy of Sciences of the United States of America. 2002;99(19):12375-80. Epub 2002/09/10. doi: 10.1073/pnas.192439399. PubMed PMID: 12218183; PubMed Central PMCID: PMC129452.

10. Leonard S, Ferooz J, Haine V, Danese I, Fretin D, Tibor A, et al. FtcR is a new master regulator of the flagellar system of *Brucella melitensis* 16M with homologs in Rhizobiaceae. Journal of bacteriology. 2007;189(1):131-41. Epub 2006/10/24. doi: 10.1128/JB.00712-06. PubMed PMID: 17056750; PubMed Central PMCID: PMC1797214.

11. Mirabella A, Yanez Villanueva RM, Delrue RM, Uzureau S, Zygmunt MS, Cloeckaert A, et al. The two-component system PrlS/PrlR of *Brucella melitensis* is required for persistence in mice and appears to respond to ionic strength. Microbiology. 2012;158(Pt 10):2642-51. Epub 2012/08/04. doi: 10.1099/mic.0.060863-0. PubMed PMID: 22859617.

12. Carrica Mdel C, Fernandez I, Marti MA, Paris G, Goldbaum FA. The NtrY/X two-component system of *Brucella* spp. acts as a redox sensor and regulates the expression of nitrogen respiration enzymes. Molecular microbiology. 2012;85(1):39-50. Epub 2012/05/16. doi: 10.1111/j.1365-2958.2012.08095.x. PubMed PMID: 22582926.

13. Dorrell N, Guigue-Talet P, Spencer S, Foulonge V, O'Callaghan D, Wren BW. Investigation into the role of the response regulator NtrC in the metabolism and virulence of *Brucella suis*. Microbial pathogenesis. 1999;27(1):1-11. Epub 1999/06/18. doi: 10.1006/mpat.1999.0278. PubMed PMID: 10373105.

14. Carrica Mdel C, Fernandez I, Sieira R, Paris G, Goldbaum FA. The two-component systems PrrBA and NtrYX co-ordinately regulate the adaptation of *Brucella abortus* to an oxygen-limited environment. Molecular microbiology. 2013;88(2):222-33. Epub 2013/03/27. doi: 10.1111/mmi.12181. PubMed PMID: 23527685.

15. Abdou E, Deredjian A, Jimenez de Bagues MP, Kohler S, Jubier-Maurin V. RegA, the regulator of the two-component system RegB/RegA of *Brucella suis*, is a controller of both oxidative respiration and denitrification required for chronic infection in mice. Infect Immun. 2013;81(6):2053-61. Epub 2013/03/27. doi: 10.1128/IAI.00063-13. PubMed PMID: 23529617; PubMed Central PMCID: PMC3676001.

16. Kim HS, Caswell CC, Foreman R, Roop RM, 2nd, Crosson S. The Brucella abortus general stress response system regulates chronic mammalian infection and is controlled by phosphorylation and proteolysis. The Journal of biological chemistry. 2013;288(19):13906-16. Epub 2013/04/03. doi: 10.1074/jbc.M113.459305. PubMed PMID: 23546883; PubMed Central PMCID: PMC3650426.

17. Galperin MY. Structural classification of bacterial response regulators: diversity of output domains and domain combinations. Journal of bacteriology. 2006;188(12):4169-82. Epub 2006/06/03. doi: 10.1128/JB.01887-05. PubMed PMID: 16740923; PubMed Central PMCID: PMC1482966.
