# Supplementary material for: LOV Histidine Kinase Modulates the General Stress Response System and Affects the virB Operon Expression in Brucella abortus
Source: PLoS One. 2015 May 19;10(5):e0124058. doi: 10.1371/journal.pone.0124058 (PMC4438053; doi:10.1371/journal.pone.0124058)
Supplement: S4 Table — (DOC) [file pone.0124058.s011.doc]

**S4 Table. Pairwise percent identities of single domain RRs associated with the GSR response.**

|  | **LovR**  ***B. abortus* 2308** | **Mext_0407**  ***M. extorquens PA1*** | **LovR**  ***E. litoralis* HTCC2594** | **LovR**  ***C. crescentus* CB15** |
| --- | --- | --- | --- | --- |
| **LovR *B. abortus* 2308** | **-** | **30.65** | **30.51** | **20.33** |
| **Mext_0407 *M. extorquens* PA1** |  | **-** | **32.20** | **22.76** |
| **LovR *E. litoralis* HTCC2594** |  |  | **-** | **21.49** |
| **LovR *C. crescentus* CB15** |  |  |  | **-** |

The aminoacid sequences of single domain RRs associated with the GSR system of four alphaproteobacteria were analyzed with Clustal-Omega2 (EMBL-EBI) : LovR from *Brucella abortus* 2308, LovR from *Caulobacter crescentus* CB15 , LovR from *Erythrobacter litoralis* HTCC2594 , and Mext_0407 from *Methylobacterium extorquens* PA1 . Pairwise percent identity is shown, which is calculated as the number of the identities between two sequences divided by the length of the alignment.

**References**

1. Sievers F, Wilm A, Dineen D, Gibson TJ, Karplus K, Li W, et al. Fast, scalable generation of high-quality protein multiple sequence alignments using Clustal Omega. Molecular systems biology. 2011;7:539. Epub 2011/10/13. doi: 10.1038/msb.2011.75. PubMed PMID: 21988835; PubMed Central PMCID: PMC3261699.

2. Foreman R, Fiebig A, Crosson S. The LovK-LovR two-component system is a regulator of the general stress pathway in *Caulobacter crescentus.* Journal of bacteriology. 2012;194(12):3038-49. Epub 2012/03/13. doi: 10.1128/JB.00182-12. PubMed PMID: 22408156; PubMed Central PMCID: PMC3370868.

3. Correa F, Ko WH, Ocasio V, Bogomolni RA, Gardner KH. Blue Light Regulated Two-Component Systems: Enzymatic and Functional Analyses of Light-Oxygen-Voltage (LOV)-Histidine Kinases and Downstream Response Regulators. Biochemistry. 2013. Epub 2013/06/29. doi: 10.1021/bi400617y. PubMed PMID: 23806044.

4. Metzger LC, Francez-Charlot A, Vorholt JA. Single-domain response regulator involved in the general stress response of *Methylobacterium extorquens*. Microbiology. 2013;159(Pt 6):1067-76. Epub 2013/04/19. doi: 10.1099/mic.0.066068-0. PubMed PMID: 23596318.
